# Single- and multi-component chiral supraparticles as modular enantioselective catalysts

**DOI:** 10.1038/s41467-019-12134-4

**Published:** 2019-10-23

**Authors:** Si Li, Juan Liu, Naomi S. Ramesar, Hendrik Heinz, Liguang Xu, Chuanlai Xu, Nicholas A. Kotov

**Affiliations:** 1 State Key Lab of Food Science and Technology, International Joint Research Laboratory for Biointerface and Biodetection, School of Food Science and Technology, Jiangnan University, Wuxi, 214122 Jiangsu People’s Republic of China; 2 Department of Chemical Engineering, University of Michigan, Ann Arbor, MI 48109 USA; 3 Biointerfaces Institute, University of Michigan, Ann Arbor, MI 48109 USA; 4 Department of Chemical and Biological Engineering, University of Colorado Boulder, Boulder, Colorado, USA; 5 Department of Materials Science, University of Michigan, Ann Arbor, MI 48109 USA; 6 Macromolecular Science and Engineering, University of Michigan, Ann Arbor, MI 48109 USA

**Keywords:** Asymmetric catalysis, Photocatalysis, Synthesis and processing

## Abstract

Nanoscale biological assemblies exemplified by exosomes, endosomes and capsids, play crucial roles in all living systems. Supraparticles (SP) from inorganic nanoparticles (NPs) replicate structural characteristics of these bioassemblies, but it is unknown whether they can mimic their biochemical functions. Here, we show that chiral ZnS NPs self-assemble into 70–100 nm SPs that display sub-nanoscale porosity associated with interstitial spaces between constituent NPs. Similarly to photosynthetic bacterial organelles, these SPs can serve as photocatalysts, enantioselectively converting *L-* or *D-*tyrosine (Tyr) into dityrosine (diTyr). Experimental data and molecular dynamic simulations indicate that the chiral bias of the photocatalytic reaction is associated with the chiral environment of interstitial spaces and preferential partitioning of enantiomers into SPs, which can be further enhanced by co-assembling ZnS with Au NPs. Besides replicating a specific function of biological nanoassemblies, these findings establish a path to enantioselective oxidative coupling of phenols for biomedical and other needs.

## Abstract (web summary)

Nanoscale biological assemblies play crucial roles in all living systems and display a variety of chemical functionalities. Here the authors show that it is possible to replicate some of the biochemical functions in similarly-sized assemblies made from inorganic nanoparticles.

## Introduction

Nanoscale biological assemblies are composed of a diverse spectrum of (bio)organic components, which are common in all living systems^1,2^. They have characteristic dimensions ranging from 50 to 500 nm and are exemplified by viral capsids^3^, endosomes^4^, exosomes, carboxysomes^5^, azurophilic granules^6^, light-harvesting bacterial organelles^7^, cellular vesicles^8^, intraluminal vesicles, stress granules^9^, and a wide spectrum of intracellular membraneless compartments. These self-contained biological spheroids, chemically related to micelles, are responsible for numerous intracellular and extracellular functions, including high-efficiency site-specific transport^2,8^, cargo protection^3^, signaling^10^, catalysis^11^, light absorption, biomolecular filters, autophagy^9^, and protein folding^12,13^. Nanoassemblies with chemically similar functions are needed for different technological areas, exemplified by drug delivery, medicine, biotechnology, solar fuels, and CO_2_ reduction. Bioinspired nanoscale assemblies can be produced from human-made components, including recombinant proteins and amphiphilic biomacromolecules^14,15^. Similar spheroidal structures can also be made using DNA nanotechnology^16^. However, the ex-vivo use of all these nanoassemblies requires the negotiation of both the benefits and problems related to biomolecular building blocks. One of the problems is their stability in reactive media that diminishes the longevity of proteins^17^, RNA^18^, and DNA^19^. Since inorganic nanoparticles (NPs) have essentially the same ability to spontaneously assemble into complex superstructures^20^, it is possible to recreate some of these assemblies and functions from inorganic components that are robust and inexpensive, which is explored in this study.

Inorganic constructs that are conceptually similar to nanoscale assemblies from (bio)organic subunits are represented by supraparticles (SPs), formed from 50–500 individual NPs^21,22^ Similar to micelles, they self-assemble because of the interplay between short-range attractive and long-range repulsive forces associated with the constituent building blocks^21,23^. Gradually increased electrostatic repulsion of SPs terminates the attachment of new NPs when a certain threshold is reached; this leads to self-limited growth and thus size uniformity. SPs display sizes and geometries reminiscent of biological nanoassemblies, and can adopt a range of morphologies from fairly simple structures, such as nanospheres^24^ or nanoshells^25^ to fairly sophisticated left- and right-handed helices^26^. SPs made of NPs are structurally flexible, enabling their modular engineering from different constitutive units, which are evidenced by the inclusion of biological nanoscale components into the structures of SPs.

Among the many known functions of biological nanoassemblies, catalysis is perhaps the most common one. Chiral catalysis takes place in many of these nanoscale “organs”, including chloroplasts^27^, endoplasmic reticulum^28^, and endosomes^4^. Following the ever-growing demand for chiral intermediates^29^ and novelty of chiral NPs, it will be important to investigate their advantages compared to homogeneous chiral catalysts^30^. Several recent studies on NPs as heterogeneous chiral catalysts have demonstrated their synthetic simplicity and catalytic activity^31–34^. However, stereo-selectivity in these nanoscale constructs is provided by complex metalorganic ligands, which leads to vulnerability of these catalysts to ligand replacement, oxidation, and cross-linking. In a way, inorganic core of NPs served in these studies serves mostly as a carrier rather than as the source of chiral bias. Conversely, individual NPs with chiral ligands that are short and simple fail to display observable enantioselectively^35^.

SPs are especially desirable as chiral catalysts because they can be constructed from metal, ceramic, and semiconductor NPs^36–38^, integrating different components into one structural unit optimized for stability, enantioselectivity, and energy efficiency. Here we show that SPs constructed from chiral NPs display catalytic activity and enantioselectivity for the photoinduced oxidation of tyrosine (Tyr) known to be one of the difficult reactions for chiral catalysis. The photocatalytic processes in inorganic SPs mimic Tyr dimerization in photosynthetic bacterial organelles^39^, demonstrating functional replication of biological nanoscale assemblies. Furthermore, enantioselectivity for Tyr–Tyr has not been reported due to lack of efficient catalysts^40,41^ while asymmetric oxidative coupling of other phenols was shown to be difficult to achieve even with sophisticated metal-organic catalysts^42,43^. The catalytic functions in these assemblies can be optimized by taking advantage of SP modularity, combining NPs with different chemical and optical properties.

## Results

### Key problems of nanoassemblies for chiral catalysis

While numerous chiral NPs and their assemblies have been reported over the last two decades, these studies were primarily focused on understanding and maximizing chiroptical activity^33–35,44,45^. When designing NP assemblies to display chiral bias, one encounters the fundamental problem of the mismatch in scales between the chiral geometries characteristic of small molecules needed for pharmaceuticals, liquid crystals, chiral polymers, and other needs versus the chiral shapes of NPs. For example, in the classical case of tartrate, only five atoms constitute the chiral atomic group with a dimension of only a few angstroms, whereas the number of atoms in chiral NPs and constructs thereof can exceed tens of thousands, with dimensions from a few nanometers to 100 nm. Additionally, NPs always have structural imperfections and variability in their atomic and molecular scales that inevitably lead to difficulties with the realization of fine angstrom-scale stereochemical control typically expected for chiral catalysts, albeit this may not be always needed.

We addressed these problems using SPs formed from semiconductor NPs as the basic structural modules^25^. The interstitial spaces between NPs in these nanospheroids are subnanometer in width and are comparable to the dimensions of small molecules serving as catalytic substrates. However, there is a tendency for NPs to form assemblies with nearly epitaxial merging of crystal lattices^26,46,47^. While useful in electronics^48^ and energy storage applications^49^, this is detrimental to chiral catalysis because it eliminates the SP’s chirality in the nanoscale pores. Therefore, NPs with relatively high recrystallization energies and interatomic bonds with fairly strong covalent character are preferred.

### Assembly of chiral SPs

**Fig. 1:**
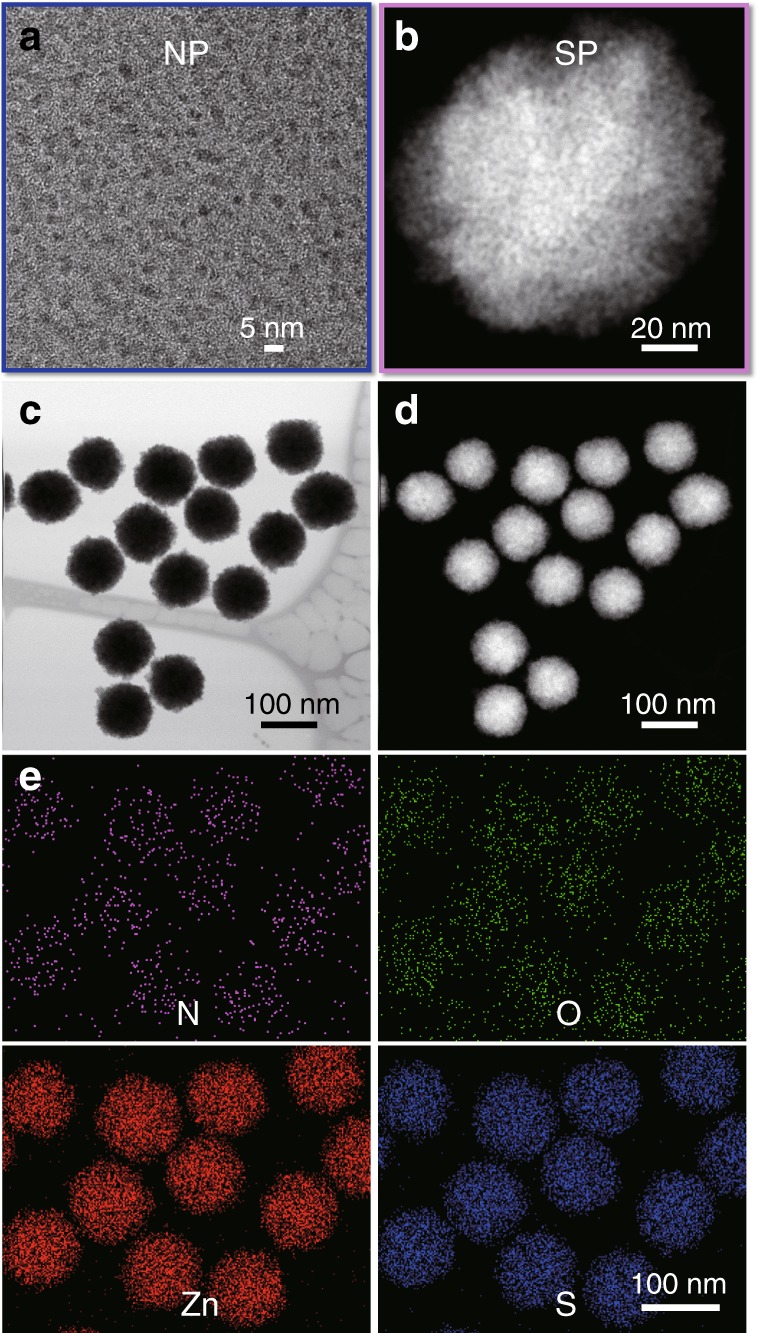
Pen-stabilized chiral NPs and SPs. **a** TEM image of l-Pen-stabilized ZnS NPs. **b** Magnified HAADF-STEM image of l-ZnS SPs that assembled from the chiral NPs shown in **a**. **c** HAADF-STEM image and **d** Bright-field-STEM image of l-ZnS SPs (100 ± 4 nm) with large scale. **e** Elemental mapping images of nitrogen, oxygen, zinc, and sulfur ZnS SPs

Assembly of chiral SPs can be accomplished by assembling ZnS NPs with an average diameter, *d*_TEM_ = 3 ± 0.7 nm, stabilized by l- or d-penicillamine (Pen) (Fig. 1a); when needed, the *rac*-form of Pen was also utilized, resulting in NPs of similar size and polydispersity. The synthesized chiral SPs will be referred to as l-, d-, or *rac-*SPs, depending on the chirality of the surface ligand tethered to the NPs. Unlike optical centers formed by *sp*^*3*^ hybridized carbon atoms, chiral NPs and their assemblies have multiple scales of mirror asymmetry^29^. Thus, l*-*, d*-*, or *rac-*notations refer only to the preparative methods rather than to geometrical attributes of the nanoassemblies.

Pen-ZnS NPs were synthesized and assembled into SPs by incubation at 95 °C for 3–6 h (see Methods). The individual ZnS NPs exhibited irregular morphology as seen from the high-resolution transmission electron microscopy (HR-TEM) images (Supplementary Fig. 1). Association of NPs is accompanied by the concomitant increase of the absolute value of electro-kinetic zeta-potential (*ζ*) (Supplementary Fig. 2). The point where it reaches a plateau corresponds to the formation of SPs, and for Pen-ZnS NPs, this occurs in 3 h, when *ζ* reaches −47 mV. The resulting SPs displayed a spherical morphology with diameters determined by TEM and dynamic light scattering (DLS) of *d*_TEM_ = 100 ± 3 nm and *d*_DLS_ = 105 ± 10 nm, respectively (Fig. 1b−d, Supplementary Figs. 3 and 4). They also showed high monodispersity with standard deviation <6%, which indicates the quasi-equilibrium state of the SPs. Elemental mapping confirmed the composition of SPs, indicating the presence of ZnS and Pen (Fig. 1e). The presence of Pen ligands in ZnS SPs was also confirmed by the Fourier-transform infrared spectroscopy (FTIR) and Raman spectra (Supplementary Figs. 5 and 6). The typical peaks at 3000–3300 cm^−1^ are attributed to the stretching vibration of N–H of Pen attached to the surface of ZnS NPs. The peaks at 1450–1700 cm^−1^ and 2200–2450 cm^−1^ are attributed to C–H bending and S–H stretching vibrations of Pen. The peaks associated with S–H bonds could not be observed for Pen-ZnS SPs as expected, due to the formation of S–ZnS bonds.

Individual NPs could be identified in the assembled SPs by high-angle annular dark-field scanning TEM (HAADF-STEM) images (Fig. 1b, Supplementary Fig. 9) and high-resolution STEM images (Supplementary Figs. 7and 8). The pore size of the SPs are below 2 nm (Supplementary Fig. 10b), which are comparable with the sizes of reactive centers on many enzymes^50^. The X-ray diffraction (XRD) spectra for SPs revealed [0036], [110], [1136], [1211] peaks of ZnS in SPs (Supplementary Fig. 11).

### Chirality of assembled SPs

**Fig. 2:**
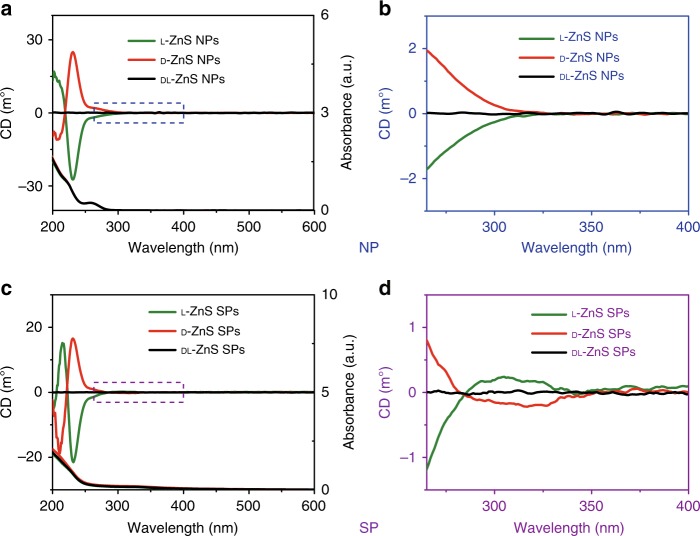
CD spectra of Pen-stabilized ZnS NPs and ZnS SPs. **a** CD spectra and UV spectra of ZnS NPs with Pen surface ligands of different handedness. **b** Enlarged CD spectra of ZnS NPs from 265 to 400 nm. **c** CD spectra and UV spectra of ZnS SPs with Pen surface ligands of different handedness. **d** Enlarged CD spectra of ZnS SPs from 265 to 400 nm

ZnS NPs with l- and d-Pen as surface ligands displayed circular dichroism (CD) peaks at 215 and 240 nm (Fig. 2a, b). Compared with the CD spectra of Pen alone (Supplementary Fig. 12), a new CD peak appeared from 240 to 300 nm; this may be attributed primarily to electronic hybridization between the molecular orbitals of NPs and those of chiral ligands. The local distortions of the ZnS surface due to attachment of Pen ligands may contribute to the CD spectra, too.

Assemblies of individual NPs into ordered structures may exhibit CD signatures of the chiral geometries of NPs that may or may not possess a distinctively chiral shape, such as a helix. Unlike the individual NPs, the chiroptical activity of the prepared SPs included an additional band in the 280–340 nm region. Note that the energies of electronic state developing as a result of hybridization of chiral states from amino acid residues attached to the NP surface are dependent on the environment. They are different for the individual NPs and that in the interstitial spaces and thus are located at longer wavelengths. One can also expect that CD peak for such electronic states will be broad as they are, indeed, in Fig. 2d.

### Assembly of multi-component chiral SPs

**Fig. 3:**
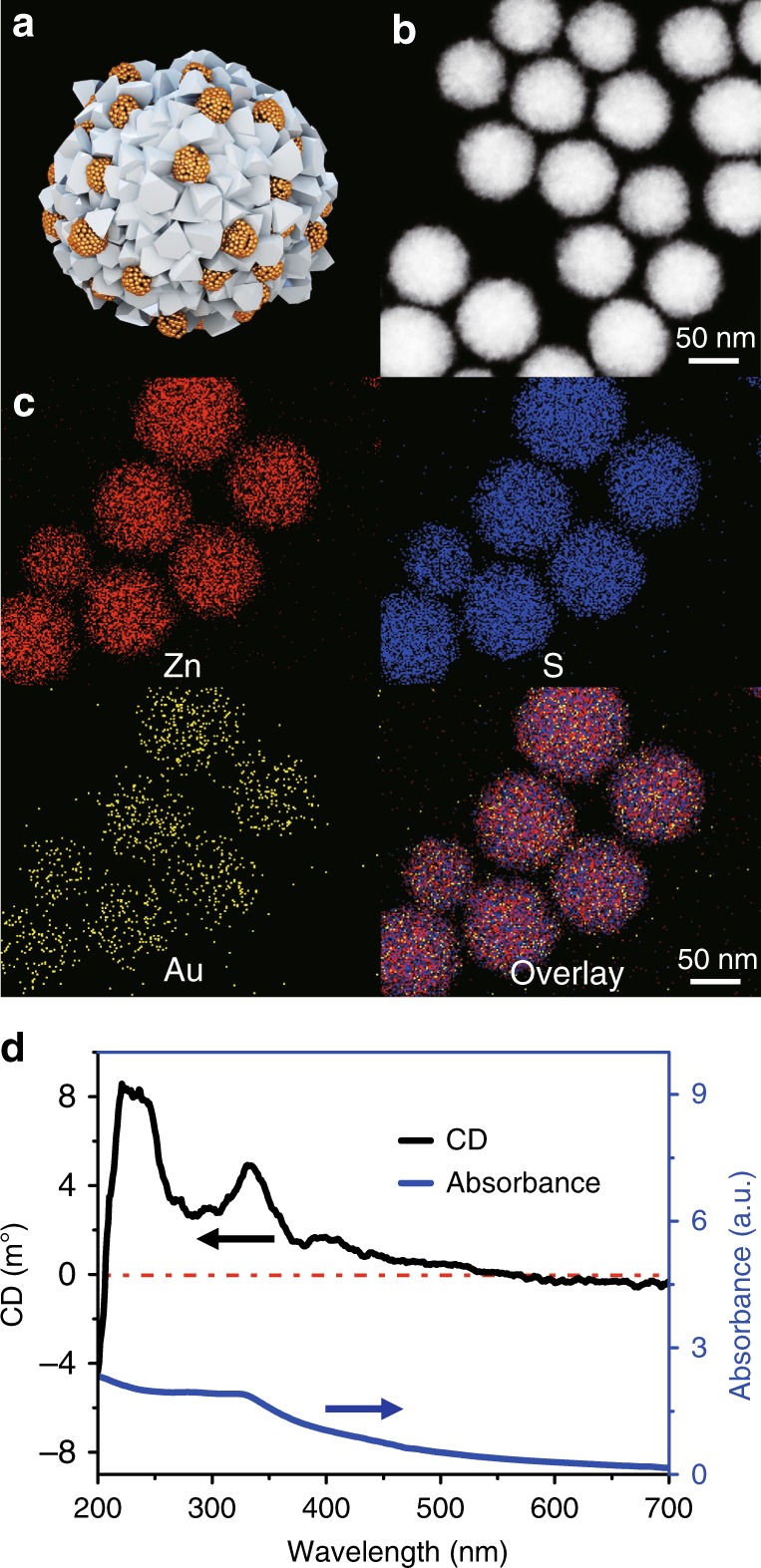
Morphology and spectra characterization of chiral ZnS–Au SPs. **a** Model of multi-component l-ZnS–Au SPs. **b** HAADF-STEM images of multi-component l-ZnS–Au SPs. **c** Elemental mapping of l-ZnS–Au SPs of zinc, gold, and sulfur, respectively. **d** CD and UV spectra of multi-component l-ZnS–Au SPs

Similar to other terminal assemblies, SPs can be made from several different NPs so that they may offer a combination of different functionalities. For instance, the co-assembly of NPs from metals and semiconductors is a simple process that results in the combination of NPs with plasmonic and excitonic properties, which should facilitate photocatalytic processes. Gold NPs carrying glutathione (GSH) units as surface ligands were chosen as complementary unimers to the Pen-stabilized ZnS NPs for the assembly of plasmon–exciton SPs because of their similarity in size. When GSH-Au NPs with diameters of 2 ± 0.3 nm (Supplementary Fig. 13) were added to a dispersion of ZnS SPs and incubated for 3 h at 95 °C, hybrid ZnS–Au SPs containing both types of constituent blocks were obtained (Fig. 3a). The *ζ*-potential of the multi-component SPs stabilized at a high value of ~−44 mV after 4 h of incubation, indicating the successful formation of SPs (Supplementary Fig. 14). Note that the *ζ*-potential characteristic of the completed self-assembly process is nearly identical to that found for ZnS nanospheroids (Supplementary Fig. 2). The average diameter of ZnS–Au SPs was *d*_TEM_ = 75 ± 3 nm (Fig. 3b; Supplementary Fig. 15), which was about 25% smaller than the diameter of single-component nanoassemblies. Elemental mapping confirmed the presence of gold NPs in these SPs (Fig. 3c). ZnS–Au SPs displayed CD signals from 200 to 550 nm (Fig. 3d). The polarity of chiroptical activity at 220 nm for l*-*ZnS–Au, that is, those where ZnS NPs were made with l*-*Pen, was opposite compared with the CD spectra of single-component ZnS SPs (Fig. 2d) and GSH-Au NPs (Supplementary Fig. 13b). The polarity reversal may have multiple physical origins^29^ and likely represents a convolution of various effects. It may be attributed to both strong chemical (such as hydrogen bonding between the surface ligands) and physical (such as plasmon–exciton hybridization) interactions between ZnS and Au NPs in the nanoassemblies.

### SPs as modular photocatalysts

The chirality of SPs can potentially endow them with enantioselectivity in catalysis. Realization of enantioselective photocatalysts would be particularly promising because the photoinduced reactions require both optical activity and robustness of inorganic NPs to prevent their photodecomposition. The conversion of the amino acid Tyr, which can be monitored using its strong photoluminescence (PL) peak at 306 nm, was used as a chiral catalytic substrate. Furthermore, Tyr can be photo-oxidized to dityrosine (diTyr)^51–55^ or dihydroxy phenylalanine (DOPA)^53^, with characteristic PL peaks at 414 and 320 nm (Supplementary Fig. 16), respectively. The realization of asymmetric photo-oxidative coupling of Tyr to diTyr would be particularly impactful from the standpoints of both chiral catalysis and pharmaceutical intermediates because enantioselectivity for such reactions was so far challenging to realize^40–42^.

**Fig. 4:**
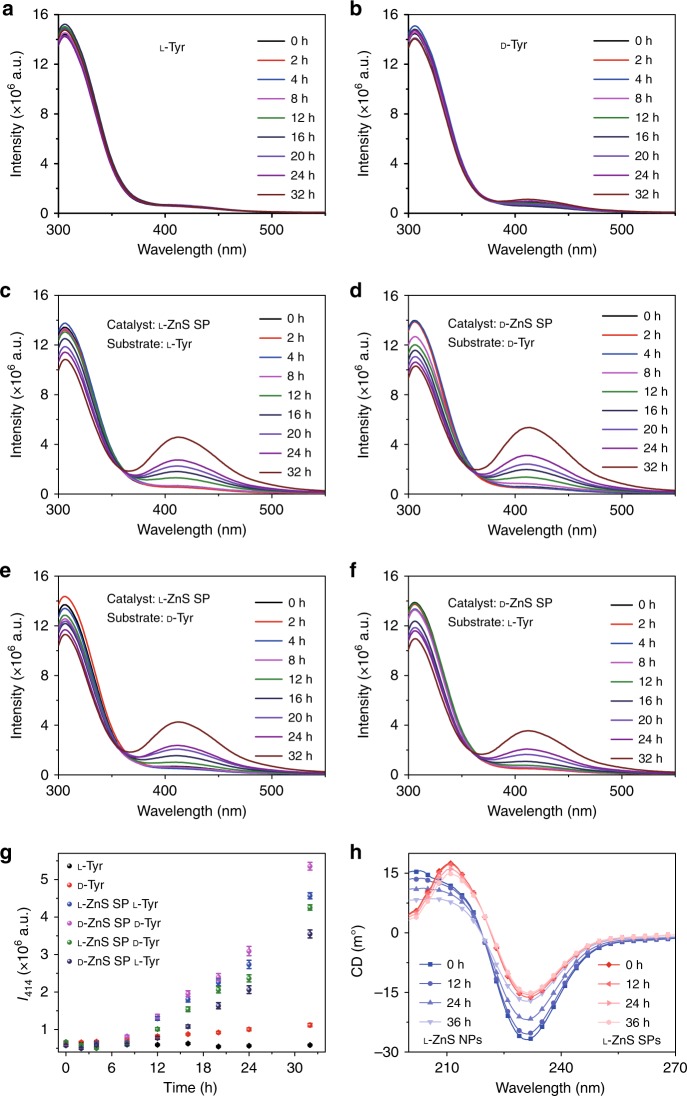
Catalisis and stability of ZnS SPs for photoinduced oxidative coupling of l-Tyr and d-Tyr. Fluorescence spectra of l-Tyr or d-Tyr after **a** l-Tyr, **b** d-Tyr, **c** l-Tyr with l-ZnS SPs, and **d** d-Tyr with d-ZnS SPs. **e** l-Tyr with d-ZnS SPs and **f** d-Tyr with l-ZnS SPs being illuminated with different periods of time in the absence or presence of ZnS SPs. **g** The dependence of PL intensity at 414 nm on the time of photocatalytic reaction with ZnS SPs of different handedness of the catalyst and the substrate. **h** CD spectra of the mixtures of ZnS NPs and ZnS SPs after UV illumination (115 V, 50/60 Hz, 0.16 A) for different times

The photocatalytic reactions in all experimental series were initiated by illumination at pH 8.0 with UV light at 315−400 nm, which was efficiently absorbed by ZnS and ZnS–Au SPs (Figs. 2c and 3d). Illumination of Tyr without SPs yielded a nearly negligible change in PL spectra (Fig. 4a, b). When ZnS SPs were added to the Tyr solution, a strong increase in PL intensity was observed at 414 nm, which is characteristic of the accumulation of diTyr (Fig. 4c–f)^52,54^. DiTyr formation was accompanied by a decrease of PL intensity at 306 nm as expected considering the consumption of Tyr. While diTyr was the main product of the photocatalytic conversion of Tyr, the formation of DOPA may also be expected, which explained the red-shift of the emission peak at 306 nm in Fig. 4 as the photolysis proceeded.

Analysis of temporal profiles of diTyr emission, *I*_414_(*t*), allowed us to evaluate the chiral preferences of the SP catalysts. ZnS SPs carrying l*-*Pen preferentially catalyzed l*-*Tyr, while SPs made from d-Pen preferentially catalyzed d-Tyr (Fig. 4g). The degree of enantioselectivity can be associated with preferential partitioning of enantiomers of Tyr into the SPs, which was confirmed by an enantioselective adsorption of Tyr enantiomers after exposure of *rac*-Tyr solution to l- or d*-*ZnS SPs. The effect of preferential adsorption of enantiomers on chiral substrates and phases was observed for some other systems^44,56–58^, but, to the best of our knowledge, not for SPs or any other nanoscale assemblies of NPs. Calibration of the CD spectra with respect to the total concentrations of each enantiomer (Supplementary Figs. 17 and 18) yields preferential partitioning of l-Tyr and d-Tyr into l*-* and d-ZnS-SPs as 38.4 ± 1.8% and 36.3 ± 2.4% over the other isomer, respectively, while that of l*-* and d-ZnS NPs was 11.5% ± 3.1% and 15.1% ± 3.4% over the other isomer. Thus, the enantioselective recognition was vividly enhanced via assembling chiral NPs into SPs. The chiral preferences of SP–substrate interactions match those observed in photocatalysis, which is consistent with the enantioselective physisorption of substrates with NPs and SPs (Fig. 4g). The individual ZnS NPs with l- or d-ligands did not show obvious catalytic preference for enantiomeric Tyr, and the catalytic activity is also lower than that of the SPs (Supplementary Fig. 19). Both the ZnS NPs and ZnS SPs retain excellent stability in the course of photocatalysis (Supplementary Figs. 20 and 21). Importantly, the photochemical stability of chiral ligands on SPs is markedly better than that on NPs. In fact, no decomposition of Pen was observed in SPs after intense illumination for 36 h (Fig. 4h).

**Fig. 5:**
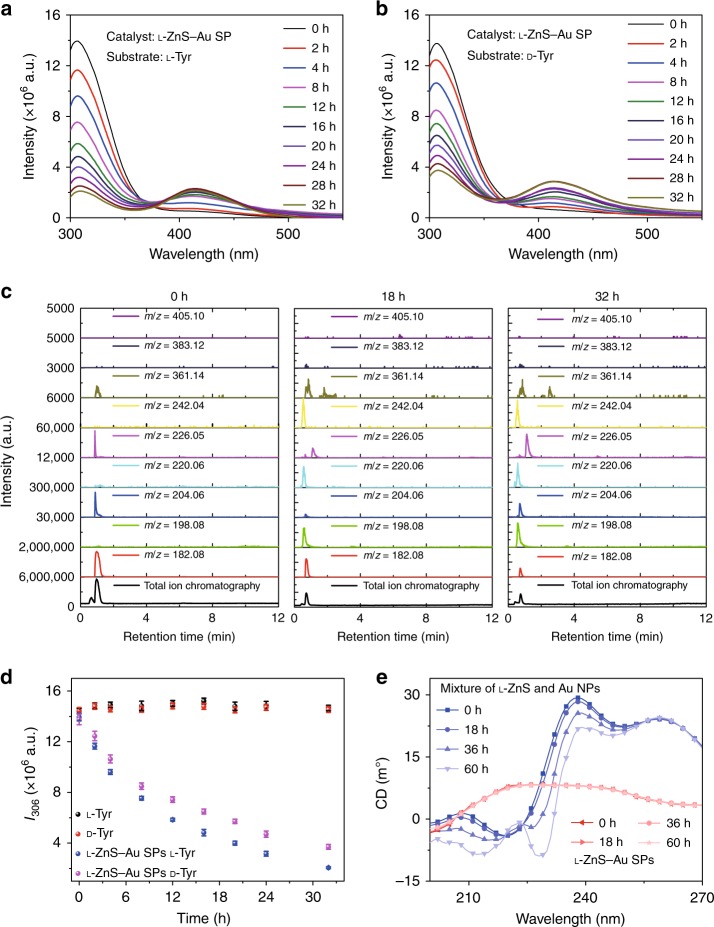
Catalysis and stability of ZnS–Au SPs for photoinduced oxidative coupling of l-Tyr and d-Tyr. Fluorescence spectra of l-Tyr or d-Tyr after **a** l-Tyr with l-ZnS–Au SPs, **b** d-Tyr with l-ZnS–Au SPs being illuminated with different periods of time in the presence of ZnS–Au SPs. **c** Extracted ion chromatography (EIC) of l-Tyr and Tyr-related products of l-ZnS–Au SP l-Tyr samples obtained at 0, 18, and 32 h, *m/z* *=* 405.10, 383.12, and 361.14, and *m/z* *=* 242.04, 220.06, and 198.08 are attributed to DOPA-Na + Na^+^, DOPA + Na^+^, and DOPA + H^+^ and diTyr-Na + Na^+^, diTyr + Na^+^ and diTyr + H^+^, respectively. **d** The dependence of PL intensity at 306 nm on photocatalytic reaction time with l*-*ZnS–Au SPs for different Tyr enantiomers. **e** CD spectra of the mixtures of ZnS and Au NPs, ZnS–Au SPs, after UV illumination (115 V, 50/60 Hz, 0.16 A) for different times

When Tyr was illuminated in the presence of ZnS–Au SPs, the fluorescence intensity of the 306 nm band decreased dramatically during the illumination (Fig. 5a, b), which indicated a large enhancement of the efficiency of photocatalytic conversion of Tyr after incorporation of Au NPs. Note, however, that the rise of the 414 nm peak associated with diTyr was smaller in ZnS–Au than in ZnS SPs (Figs. 4c–f and 5a, b), pointing to the simultaneous enhancement of diTyr conversion into non-luminescent products.

As a result of the higher catalytic activity of ZnS–Au SPs, weaker PL signature of diTyr, and greater complexity of the products than in single-component SPs, the chemical composition of products of photocatalysis were investigated using additional analytical techniques. High-performance liquid chromatography–mass spectrometry (HPLC-MS) confirmed the efficiency of the photoinduced oxidative coupling of Tyr by ZnS–Au SPs. The peaks in extracted ion chromatography (EIC) provided spectra tracings that concomitantly verified the conversion of Tyr to diTyr and DOPA (Fig. 5c). The gradual stabilization of the EIC peaks corresponding to diTyr and DOPA for the reaction time from 18 to 32 h confirmed the ability of multi-component SPs to subsequently oxidize diTyr and DOPA, which was not the case for single-component SPs.

Similarly to ZnS SPs, the temporal dependence of PL intensity at 306 nm revealed that l-ZnS–Au SPs preferentially catalyzed the photo-oxidation of l-Tyr over d*-*Tyr (Fig. 5d). The association of enantioselectivity of the photocatalysis with preferential partitioning of a specific enantiomer was confirmed for the multi-component SPs. When incubated with *rac*-Tyr, l-ZnS–Au SPs prefer to adsorb l-Tyr in the amount 44.2 ± 1.7% greater than that for d-Tyr (Supplementary Figs. 18 and 22). Selective adsorption of Tyr enantiomers in ZnS and ZnS–Au SPs indicates that ZnS NPs with Pen surface ligands are primarily responsible for the chiral bias of the photo-oxidation process. The improved catalytic performance of Au–ZnS SPs can be attributed to more facile separation of photogenerated electrons and holes and the reduced band gap of ZnS. The photogenerated electrons prefer Au surface rather than ZnS. The holes, however, will mainly stay on the ZnS nanoparticles, which will reduce the recombination of the electron-hole pairs and enhance the catalytic efficiency.

The photochemical stability of chiral ligands on Au–ZnS SPs was shown to exceed those in the dispersion of individual ZnS and Au NPs (Fig. 5e). No degradation of Pen and GSH was observed for illumination for 60 h whereas marked reduction of CD intensity of the peaks corresponding to both peptides was observed for free NPs over the same time.

There are several chemical mechanisms why the stability of the ligands would increase in SPs compared to chiral photocatalysts based on single NPs or traditional organometallic compounds. First, SPs afford utilization of short and robust chiral ligands, such as individual amino acids as molecular structures responsible for enantioselectivity, as opposed to complex and sensitive high molecular weight constructs. The higher the molecular weight of the surface ligands, the greater number of conformational degrees of freedom, and the greater the possibility of chemical damage due to photonically or thermally initiated reactions. Second, the light in SP is absorbed by a subset of NPs. These can be the NPs in the surface or Au NPs in ZnS or Zn-S–Au SPs, respectively. Unlike single NPs or the traditional chiral photocatalysts, the excitation can be efficiently transferred between several NPs due to their closeness in the SP assemblies. The resonance coupling of the excited states in SPs is advantageous for the stability of the chiral photocatalysts because on chiral ligands on the internal NPs are better protected against damage than those on the SP surface. Third, SPs represent a self-assembled hybrid composite where ZnS cores and organic ligands on their surface constitute the mineral and organic components, respectively. Multiple experimental data indicate that the organic molecules in the composite display increased resilience to oxidation, thermal decomposition, or hydrolysis^59–61^. This property represents the fundamental structural advantage of SPs as chiral photocatalysts.

### Molecular dynamics simulation

**Fig. 6:**
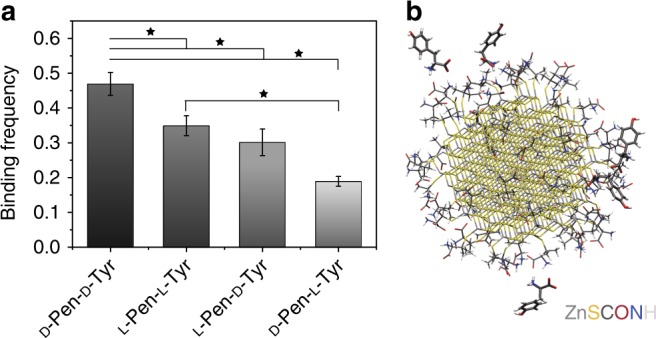
Adsorption of (l or d)-Tyr on (l or d)-Pen-ZnS NPs of 3-nm diameter from MD simulations. **a** The binding frequency decreases in the order from d-Pen-d-Tyr, l-Pen-l-Tyr, l-Pen-d-Tyr, to d-Pen-l-Tyr. Error bars indicate the standard error from 5 ns block averages during 50 ns MD simulations. **b** Snapshot of d-Tyr binding on the d-Pen-ZnS NP surface. d-Tyr molecules are represented in “Licorice” style with thicker bonds. The 3-nm d-Pen-ZnS NP is represented in “Licorice” style with thinner bonds. Binding sites are highlighted with green dotted lines and water molecules omitted for clarityANOVA analysis was used to explore significant differences of means comparison using the Fisher Test. The symbol * denotes the pairs with P value < 0.05 indicating significant difference

Molecular dynamics simulation can be a powerful tool for investigation of the atomic scale dynamics and molecular recognition properties at NP surfaces, and was applied here to obtain insight into the recognition of (l or d)-Tyr in aqueous solution on (l or D)-Pen-ZnS NPs (Fig. 6). The enantioselectivity of Pen-ZnS surfaces were evaluated using the binding frequency of Tyr enantiomers to the Pen ligands on single NPs and model nanoassemblies from four NPs (Supplementary Figure 23) during 50 ns and 20 ns, respectively, molecular dynamics simulations in explicit water at pH 7.0 (Fig. 6). Binding for both single- and four-NP systems decreases in the order d-Pen/d-Tyr > l-Pen/l-Tyr > l-Pen/d-Tyr > d-Pen-l-Tyr, which matches with the reaction rate for Tyr consumption and diTyr formation (Fig. 4g from measurements). The most preferable binding interactions occur between –COO^−^ ions in Pen and –NH_3_^+^ groups in Tyr, as well as between –NH_3_^+^ groups in Pen and –COO^−^ groups in Tyr in all four systems (Fig. 6b). The binding involves ion pairing and hydrogen bonds. Once two binding contacts simultaneously form between a Pen ligand and Tyr, Tyr will remain bound in excess of 10 ns. When only one of the binding contacts forms, the binding status is more dynamic and lasts only 1–2 ns. As the aromatic side group of Tyr approaches the bare ZnS NP surface, the –OH group may temporarily bind to the ZnS surface and disengage binding to Pen via ion pairing. Differences in binding properties for different chirality combinations of ligands and Tyr molecules are related to distinct relative orientations of the carboxylate and ammonium groups of Pen on the particle surface (inward/outward depending on d/l), as well as specific interactions between neighbor ligands. Tyr binds selectively to these chiral ligand surfaces and experiences some geometry differences in match depending on (d) or (l) configuration. In addition, the binding competition between the charged groups in Tyr (–NH_3_^+^ and –COO^−^) with Pen ligands and the aromatic side groups in Tyr with uncovered areas of the ZnS NP surface demonstrates that the pH value and the density of surface ligands can affect the binding affinity. Diffusion rate of L-Tyr in the center of four-NP assembly was found to be 0.22±0.05 and 0.34±0.12 A/ns for D- and L-Pen-ZnS models, respectively. Concurrently, the diffusion rates of L-Tyr on the sides of the nanoassembly were much faster - 2.2±0.6 and and 1.8±0.5 A/ns for the same models. This observation supports the attribution of experimentally observed enhancement of both catalytic activity and enantioselectivity in SPs vs. NPs to extended residence time of substrate inside the catalytic particle.

## Discussion

Single- and multi-component SPs with size and uniformity comparable to biological nanoassemblies were assembled from chiral semiconductor and metallic NPs mimicking both structure and functions of their biological analogs, for instance photosynthetic organelles of bacteria and, to a lesser extent, redox reactions in azurophilic granules. SPs showed efficient photocatalysis of Tyr with enantioselectivity determined by the chiral preferences of l*-* and d*-*Tyr to interact with individual NPs and penetrate into the interstitial spaces between them. It needs to be pointed out that enantioselectivity of oxidative phenol coupling, which is Tyr–Tyr dimerization is representative of, is both difficult^40,41^ due to low chiral bias and significant due to variety of biomedical needs^41^. The problems with enantioselectivity are particularly noticeable for low molecular weight phenols as opposed to large molecular^42^ weight polycyclic aryls^43^. As such, optical purity of just a few percent is typically achieved even with complex metalorganic catalysts for diverse catalytic substrates^42^. In the majority of cases, however, no enantioselectivity was reported^40,41^ and poor enantioselectivity of these reactions was pointed out even for sophisticated biomimetic catalysts^42^.

Incorporation of Au NPs to realize multicomponent SPs further enhanced photocatalytic conversion of the substrates. Besides improved electron-hole separation, more efficient light absorption by the assemblies via plasmonic effects and the formation of the NP junctions can facilitate both the electron transport, while retaining enantioselectivity. Concomitantly, photodecomposition under light is an essential problem of the chiral catalysts of any chemical type. Improving the resilience of chiral photocatalysts against photodegradation is an important advancement in the field that facilitates their practical implementation.

One can expect SPs to be a convenient platform for engineering catalysts using different NPs as functional modules. SPs provide a new approach to design a family of chiral catalysts conceptually different from those considered before in the field of homogeneous and heterogeneous chiral catalysts. Permutations of different NPs that can be combined in modular SPs is nearly endless. The development of more specific and sophisticated SPs that can replicate multiple other functions of biological nanoassemblies is anticipated.

## Methods

### Reagents

Zn(ClO_4_)_2_·6H_2_O, thioacetamide, hydrogen tetrachloroaurate (III) trihydrate (HAuCl_4_·3H_2_O), glutathione, and *D*- and *L*- Tyr were purchased from Sigma-Aldrich. All chemicals were used as received. Milli-Q-deionized water was used for all the experiments.

### Characterization methods and instruments

Assemblies were characterized using a JEOL 3011 HRTEM instrument (JEOL USA, Inc.), and a JEOL2100F was employed for HAADF and BF imaging in STEM mode. Elemental analysis was performed using an EDAX accessory linked to the JEOL 2100F instrument (JEOL USA, Inc.), equipped with a UVL-56 lamp without a filter (115 V, 50/60 Hz, 0.16 A).

UV–Vis absorbance spectra were acquired using an Agilent 89090A instrument (Agilent Technologies, Santa Clara, CA, USA). CD analysis was performed using a JASCO J-815 instrument. The *ζ*-potential and size distribution were measured with a Nano ZS Zetasizer instrument (Malvern Instruments, Malvern, Worcestershire, UK). For *ζ*-potential, each sample was equilibrated for 2 min before measurement; all experiments were performed in triplicate, each measurement included 50 cycles, and a 15-s pause was included between runs.

MS analysis was performed on an Agilent 6520 Accurate-Mass Quadrupole Time-of-Flight (Q-TOF) LC–MS instrument (Agilent Technologies, Santa Clara, CA, USA) operating in ESI^+^ ion mode. The nebulization gas was set to 500 L h^−1^ at a temperature of 332 °C, the cone gas was set to 5 L min^−1^, and the nebulizer gas pressure was 45 psi. The capillary voltage was set to 3500 V. Internal reference ion masses [M + H]^+^ at 121.05 Da and 922.01 Da were enabled, and a detection window of 200 ppm with a minimum height of 1000 counts was used. The results shown in Fig. 5c were obtained using an InfinityLab Poroshell 120 HILIC-Z HPLC column (2.1 × 50 mm, 2.7 μm internal diameter).

### Synthesis of chiral ZnS SPs

For chiral SPs, 1.36 × 10^−3^ mol of l-*,* d-, or *rac*-Pen and 1.7 × 10^−4^ mol Zn(ClO_4_)_2_·6H_2_O were dissolved in 86 mL of water, the pH was adjusted to 8.5 by adding NaOH, and the solution was degassed by argon bubbling for 30 min. A 1-mL sample of 0.2 M thioacetamide was quickly injected under vigorous stirring and the mixture was incubated at 95 °C in a reflux column system. Samples were taken at different times for measurement and characterization. Pen-stabilized SPs were obtained after 3 h of heating.

### Synthesis of chiral gold NPs

Briefly, 50 mL of 4.8 mM glutathione (GSH) solution was mixed with 300 μL of 0.5 M HAuCl_4_ under vigorous stirring. The mixture was heated at 95 °C in an oil bath to obtain chiral gold NPs. The synthesized GSH-Au NPs were purified by placing in a centrifuge at 16,200 × *g* to remove large aggregates after the reaction. The supernatant was further purified by adjusting the solution to pH 3.5, adding 1 volume of ethanol (gold NPs solution: ethanol = 1:1), isolated using a centrifuge at 5000 × *g* for 5 min, and the supernatant was discarded. Precipitates were resuspended in 20 mL of water.

### Synthesis of chiral ZnS–Au SPs

For chiral ZnS–Au SPs, 13.6 × 10^−4^ mol of l-Pen and 1.7 × 10^−4^ mol of Zn(ClO_4_)_2_·6H_2_O were dissolved in water and the pH was adjusted to 8.5. The solution was degassed by argon bubbling for 30 min. Under vigorous stirring, 1.6 × 10^−4^ mol thioacetamide dissolved in water was added and then degassed for 30 min by argon bubbling. After that, the solution was heated for 30 min at 95 °C, 6 mL of a concentrated dispersion of Au NPs was added and the volume brought to 100 mL. The pH value of the concentrated Au NPs must be adjusted to 8.5 before mixing. The resulting mixture was heated to reflux at 95 °C under flowing argon, and the final Au–ZnS SPs were obtained after 4 h of heating.

### Photocatalysis with chiral ZnS SPs and ZnS–Au SPs

Solutions of l-Tyr and d-Tyr (0.4 mg mL^−1^) were prepared, and synthesized ZnS SPs and ZnS–Au SPs were washed three times with deionized water, and then dispersed in water. Solutions of l-ZnS SPs, d-ZnS SPs, and l-ZnS–Au SPs, assembled with the same concentration of ZnS NPs, were mixed with the same volume of 0.4 mg mL^−1^ l-Tyr or d-Tyr. Control samples were prepared by mixing the same amount of water with l-Tyr or d-Tyr. Samples of l-Tyr, d-Tyr, l-ZnS SPs with l-Tyr, d-ZnS SPs with d-Tyr, l-ZnS SPs with d-Tyr, d-ZnS SPs with l-Tyr, l-ZnS–Au SPs with l-Tyr, and l-ZnS–Au SPs with d-Tyr were mixed thoroughly and the pH was adjusted to 4.7 or 8.0 before illumination. The prepared samples were illuminated with a mercury lamp for the desired time. Catalytic samples were continually stirred during illumination. SPs and solutions were separated by ultra-filtration, and filtered solutions were used for fluorescence and MS analyses. HPLC–MS measurements were performed using a 20-µL injection volume, at testing *m/z* 405.10, 383.12, 361.14, 242.04, 226.05, 220.06, 204.06, 198.08, and 182.08 that corresponded to diTyr-Na + Na^+^, diTyr + Na^+^, diTyr + H^+^, DOPA-Na + Na^+^, Tyr-Na + Na^+^, DOPA + Na^+^, Tyr + Na^+^, DOPA + H^+^, and Tyr + H^+^.

### Enantioselectivity of chiral SPs for *rac*-Tyr

l-ZnS, d-ZnS, *rac*-ZnS, and l-ZnS-Au SPs were incubated with 0.4 mg mL^−1^ *rac*-Tyr for 6 h with stirring. Sodium chloride was then added to obtain a concentration of 0.5 M in order to cause the sedimentation of SPs. As the SPs settled to the bottom, no centrifugation was needed. The supernatant from the top part of the vial was diluted with ultrapure water to avoid saturation of the detectors. The diluted samples of the supernatant were used for absorbance and CD measurements.

### Construction of molecular models and simulation setup

All-atom models of the ZnS NP, (l)-Pen, (d)-Pen, (l)-Tyr, (d)-Tyr, and water were built using Materials Studio. A NP model of 3 nm size (Zn_369_S_348_S(Ligand)_54_) was created from a multiple of the unit cell of sphalerite (ZnS) and application of a spherical cutoff. Atomic charges were assigned with charge increments of ±0.25 e per bonded neighbor, corresponding to an atomic charge of +1.0 e for Zn and −1.0 e for S in bulk ZnS, and accordingly reduced charges on the surface. Fifty-four (l or d)-Pen ligands were bound via thiol linkages to the surface and randomly spread (packing density ~1.9 nm^−2^)^62^. The protonation states of carboxylic acid groups and amine groups in (l or d)-Pen and (l or d)-Tyr were adjusted to COO^−^ and NH_3_^+^ to represent neutral conditions of pH ⋍ 7 as in experiment.

Simulations were performed for four systems, including (1) l-Pen-ZnS-l-Tyr, (2) l-Pen-ZnS-d-Tyr, (3) d-Pen-ZnS-l-Tyr, and (4) d-Pen-ZnS-d-Tyr. The dimensions for each box were the same, about 70 × 70 × 70 Å^3^, and all simulation systems were overall charge-neutral. The complete start structures consisted of one ZnS NP modified with (d) or (l) Pen, 8 (d) or (l) Tyr molecules in solution, and 10229 water molecules. The Tyr molecules were evenly placed in the box with ~ 10 Å distance from the Pen-ZnS NP.

### Force field

We employed the CHARMM-Interface force field (CHARMM-IFF) including new parameters for ZnS (Table 1)^63^ and existing CHARMM36^64^ parameters for (l or d)-Pen and (l or d)-Tyr. The new force field parameters for ZnS describe the expected amount of covalent and ionic bonding^64,65^ and reproduce the lattice parameters (5.4093 Å) and density (4.09 g cm^−3^) from XRD data (Supplementary Fig. 11) with 0.2% deviation (5.394 Å) and 0.7% deviation (4.12 g cm^−3^), respectively, at 298.15 K and 1 atm. The Lennard–Jones parameters approximated surface and interfacial energies of ZnS (not tested in detail)^66^.

**Table 1:** Forcefield parameters for ZnS (sphalerite)

I. Nonbond	Charge (e)	*σ* (pm)	*ε* (kcal mol^−1^)
Zn	+1.00	185	0.05
S	−1.00	448	0.5
II. Bond	*r*_0_ (pm)	*k*_r_ (kcal mol^−1^ Å^−2^)	
Zn–S	2.41	525	
Zn–S (ligand)	2.41	525	
III. Angles	*θ*_0_ (°)	*k*_*θ*_ (kcal mol^−1^ rad^−2^)	
Zn–S–Zn	109.5	200	
S–Zn–S	109.5	200	
S–Zn–S (ligand)	109.5	200	
Zn–S–C (ligand)	100.0	0	

Note: No dihedral potentials were necessary for bonds inside the ZnS NP (zero)

### Simulation protocol

Each system was initially subjected to 1000 time steps energy minimization to remove atomic close contacts. The configurations were then subjected to an initial 2-ns equilibration period of molecular dynamics simulation in the NPT ensemble at 298.15 K and 1.013 MPa using the Nanoscale Molecular Dynamics program (NAMD)^67^, followed by longer simulation times of 50 ns to thoroughly sample conformations and thermodynamic averages. All atoms were flexible during the simulations, the time step was 1 fs, temperature controlled by the Langevin thermostat, and a damping coefficient of 1 ps^−1^. Van-der-Waals interactions were treated with a spherical cutoff of 12 Å in the summation of pairwise Lennard–Jones interactions, and the summation of Coulomb interactions was carried out using the Particle Mesh Ewald (PME) method with a high accuracy of 10^−6^ kcal mol^−1^ throughout the equilibration and production runs.

### Analysis and calculation of binding frequency

The conformations and dynamics were visually analyzed using the Visual Molecular Dynamics (VMD) program^68^. Binding was defined as uninterrupted proximity of Tyr molecules to the Pen–ZnS NP within 5 Å for 100 ps or more. The binding frequency was calculated from the number count of binding frames, that is, the sum of the number *n*_*i*_ of Tyr molecules bound in each frame *i* over all frames *N*, relative to the total number of frames *N* and 8 Tyr molecules:

1 $${\mathrm{Binding}}\,{\mathrm{frequency}} = \frac{{\mathop {\sum }\nolimits_{{i} = 1}^{N} n_i}}{{8 \times N}}.$$

Uncertainties are given using block averages of the binding frequency over major portions of the total simulation time.

## Supplementary information

Supplementary Information

## Data Availability

The data that support the findings of this study are available from the corresponding author upon reasonable request.
