# Supplementary material for: Single- and multi-component chiral supraparticles as modular enantioselective catalysts
Source: Nat Commun. 2019 Oct 23;10:4826. doi: 10.1038/s41467-019-12134-4 (PMC6811642; doi:10.1038/s41467-019-12134-4)
Supplement: Supplementary file 1 — Supplementary Information [file 41467_2019_12134_MOESM1_ESM.pdf]

Supplementary Information

SINGLE- AND MULTI-COMPONENT CHIRAL SUPRAPARTICLES AS MODULAR ENANTIOSELECTIVE CATALYSTS

Si Li,[1,2,3] Juan Liu,[4] Naomi S. Ramesar,[2,3] Hendrik Heinz,[4] Liguang Xu,[1*] Chuanlai Xu,[1] Nicholas A. Kotov[2,3,5,6*]

[1] State Key Lab of Food Science and Technology, International Joint Research Laboratory for Biointerface and Biodetection, School of Food Science and Technology, Jiangnan University, Wuxi, Jiangsu, 214122, PRC; [2]Department of Chemical Engineering, University of Michigan, Ann Arbor, Michigan 48109, USA; [3]Biointerfaces Institute, University of Michigan, Ann Arbor, Michigan 48109, USA; [4]Department of Chemical and Biological Engineering, University of Colorado Boulder, Boulder, Colorado, USA; [5]Department of Materials Science, University of Michigan, Ann Arbor, MI, 48109, USA; [6]Macromolecular Science and Engineering, University of Michigan, Ann Arbor, Michigan 48109, USA.

[*]Corresponding authors: xuliguang2006@126.com; kotov@umich.edu

SUPPLEMENTARY METHODS

Calculation of Enantioselective Partitioning of Tyr Enantiomers into Supraparticles. The concentration of Tyr in the supernatant denoted as C_{Tyr1}, was calculated from the UV-Vis absorbance spectra in **Supplementary Fig. 17b** and **22b** using the equation $I_A = 18.200\ C_{Tyr1} - 0.0273$ (**Supplementary Fig. 18b**). The amount of Tyr (C_{Tyr2}) partitioned into SPs, which lead to the appearance of a CD signal of the supernatant solution, was calculated with the calibration equation $I_{CD} = 128.505\ C_{Tyr2} - 0.0244$ (**Supplementary Fig. 18d**) based on the data in **Supplementary Fig. 17b** and **22b**. The enantiomer selectivity for SP partitioning was calculated using the equation $V_{selectivity} = C_{Tyr2} / C_{Tyr1}$.

Chemical structures of different molecules involved in this study

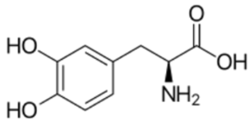

DOPA

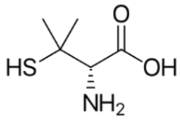

Pen

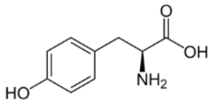

Tyr

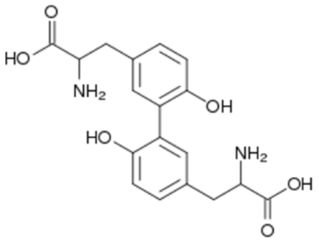

diTyr

Supplementary Figures

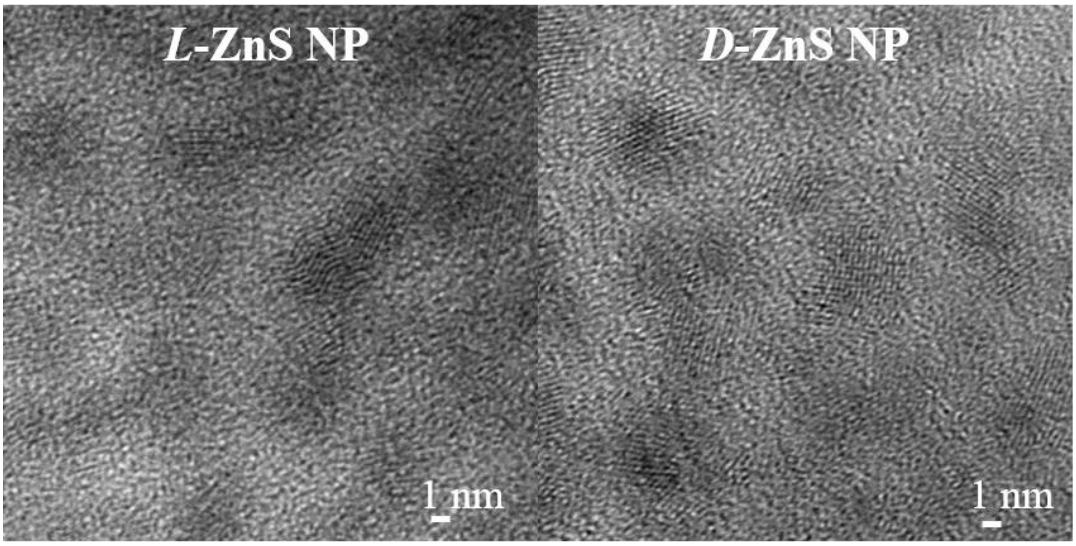

Supplementary Fig. 1 HR-TEM images of *L*- and *D*- ZnS NPs.

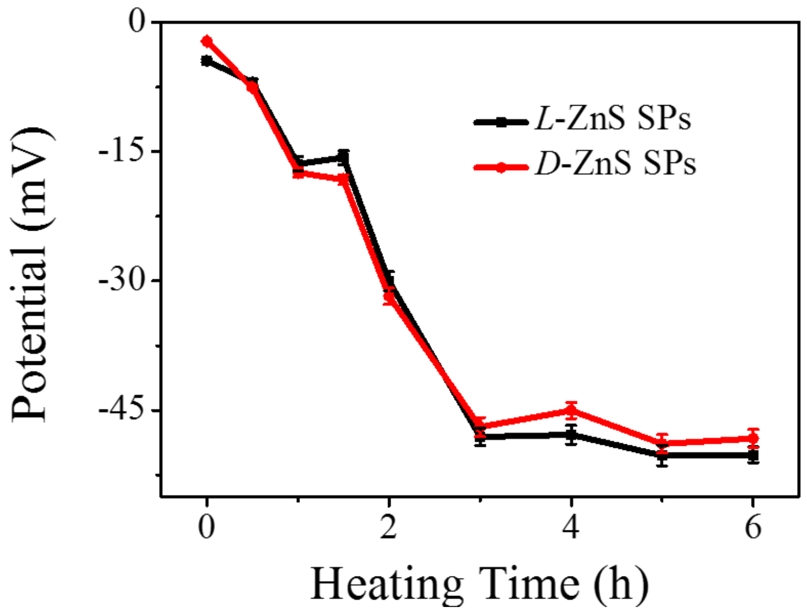

Supplementary Fig. 2 Temporal profile of ζ-potential for the assembly of *L*- and *D*-ZnS NPs. The error bars represent the standard deviation of sample measurements.

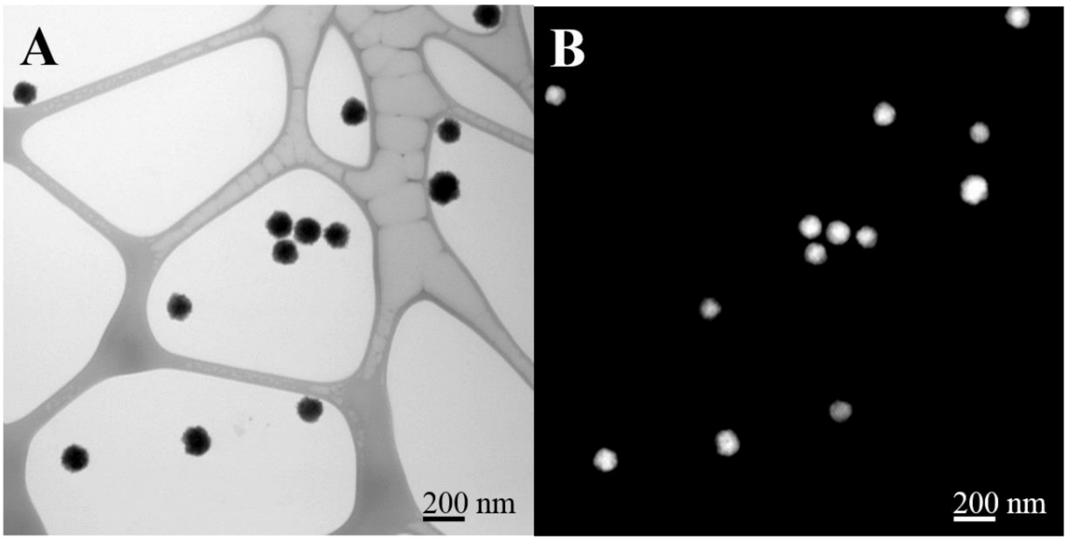

Supplementary Fig. 3 (a) Bright field- and **(b)** HAADF-STEM images of *D*-ZnS SPs.

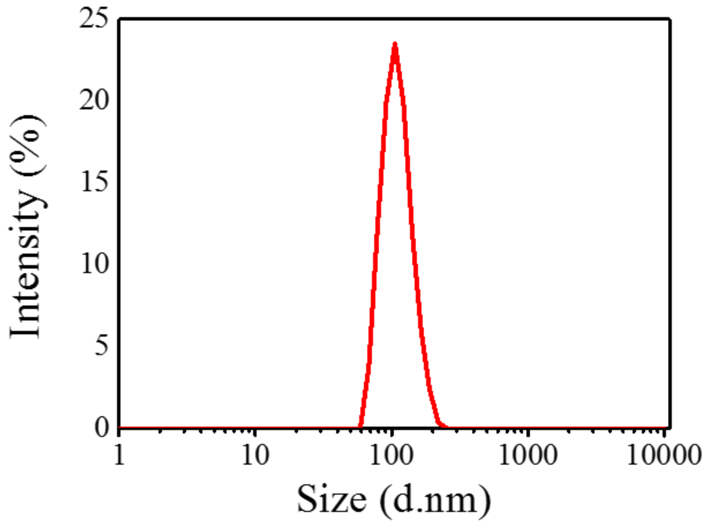

Supplementary Fig. 4 Size distribution of the self-assembled ZnS SPs by dynamic light scattering (DLS).

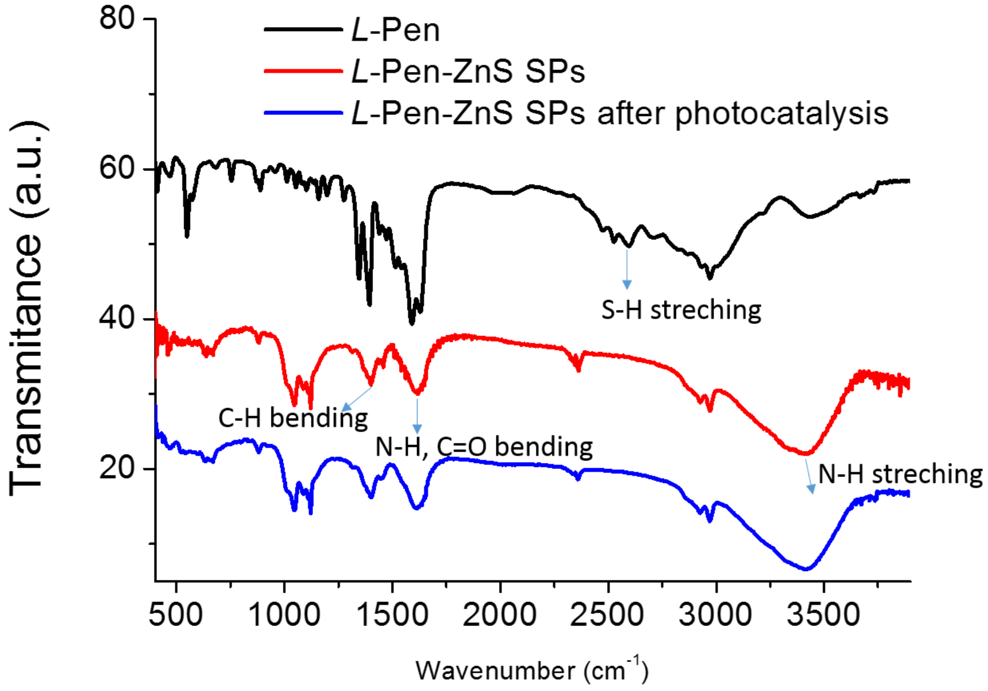

Supplementary Fig. 5 FTIR spectra (400-3900 cm^{-1}) of *L*-Pen, *L*-Pen-ZnS SPs, and *L*-Pen-ZnS SPs after photocatalysis.

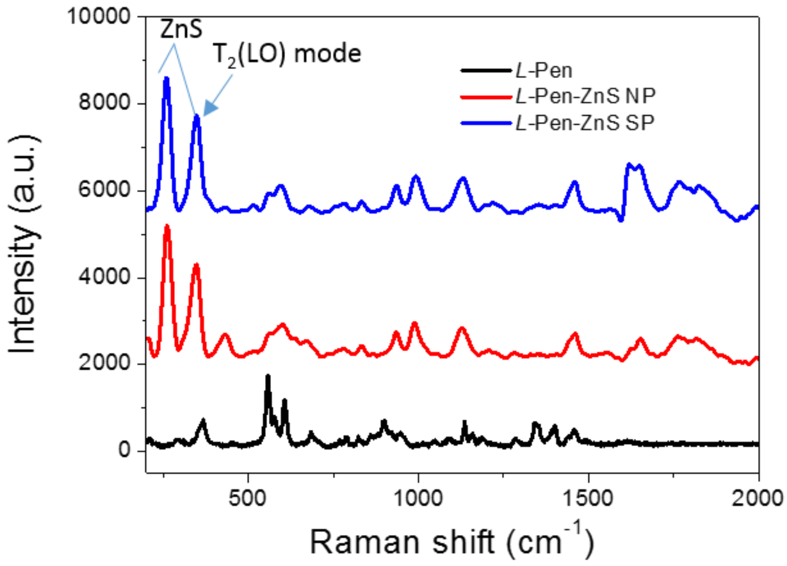

Supplementary Fig. 6 Raman spectra of *L*-Pen, *L*-Pen-ZnS NPs, and *L*-Pen-ZnS SPs.

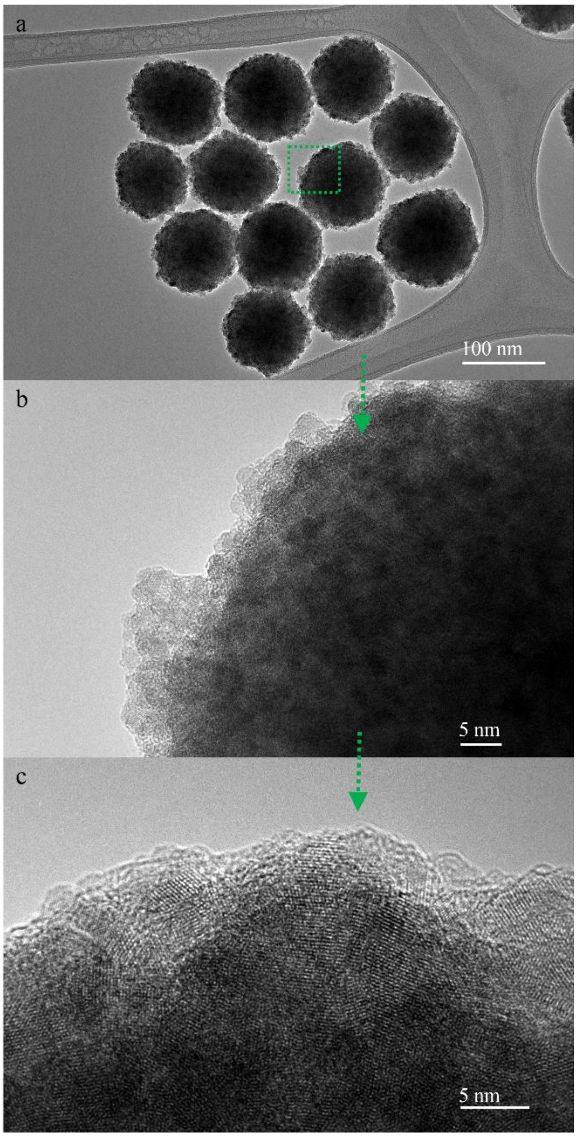

Supplementary Fig. 7 (a) TEM image of *L*-Pen-ZnS SPs. **(b)** Magnified TEM image (marked with dashed green square in Fig. S7a) of *L*-Pen-ZnS SP. **(c)** High resolution TEM image of the edge of *L*-Pen-ZnS SP.

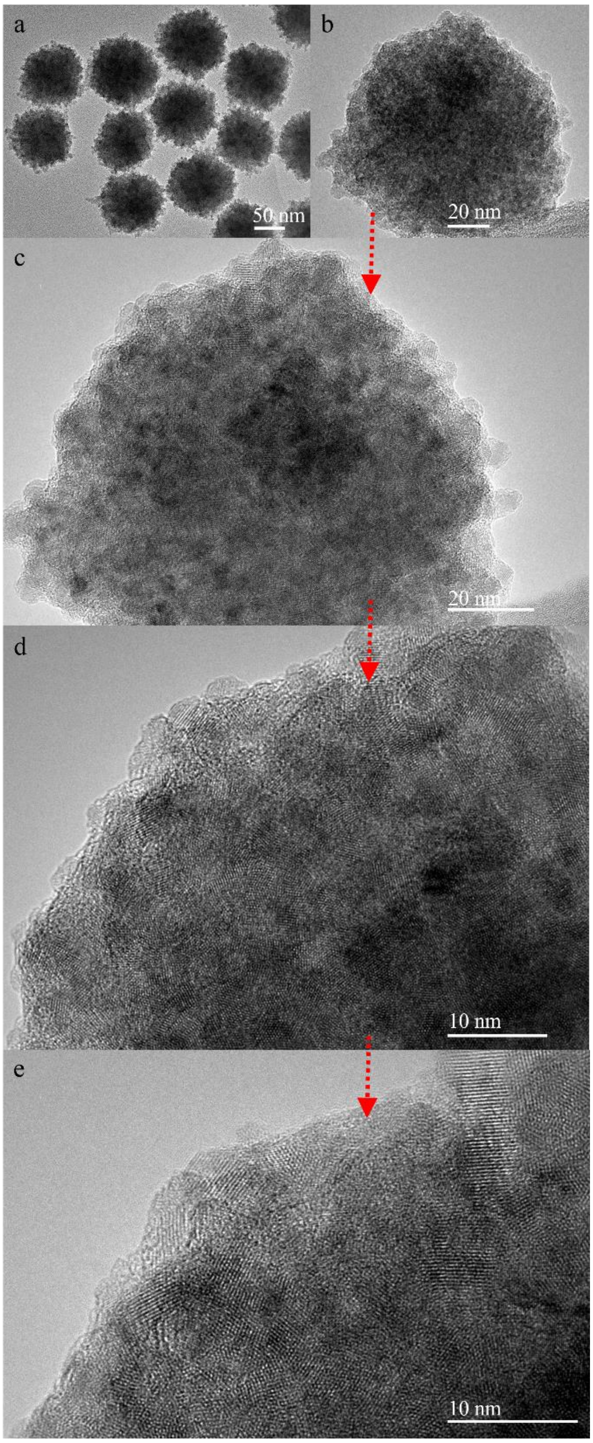

Supplementary Fig. 8 (a) TEM image of *D*-Pen-ZnS SPs. **(b)** TEM image of individual *D*-Pen-ZnS SPs. **(c)** High resolution TEM images of *D*-Pen-ZnS SPs. **(d)** and **(e)** Magnified TEM images of the edge of *D*-Pen-ZnS SP.

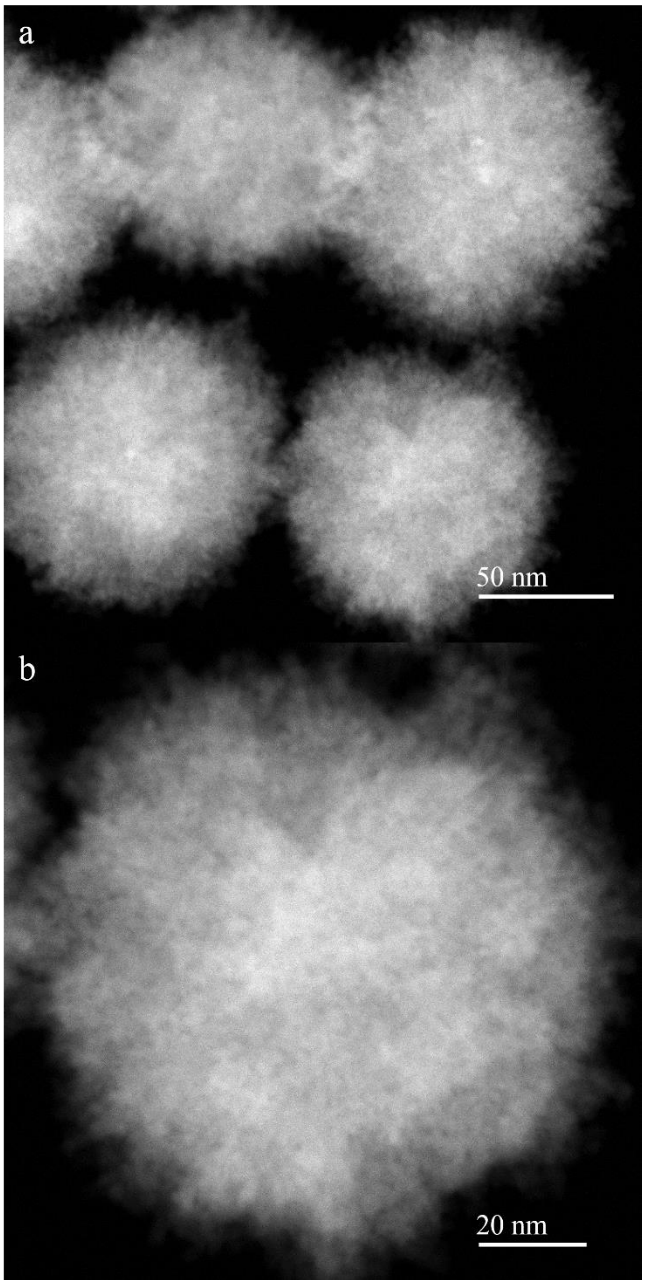

Supplementary Fig. 9 (a) HAADF-STEM images of *L*-Pen-ZnS SPs. **(b)** Magnified HAADF-STEM image of *L*-Pen-ZnS SP.

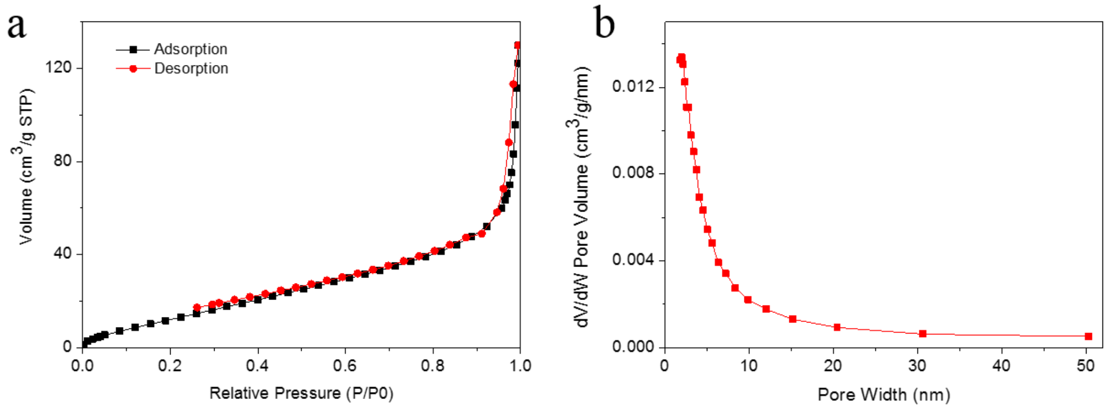

Supplementary Fig. 10 (a) N_2 adsorption-desorption isotherm and **(b)** corresponding pore size distribution of the *L*-Pen-ZnS SPs.

a

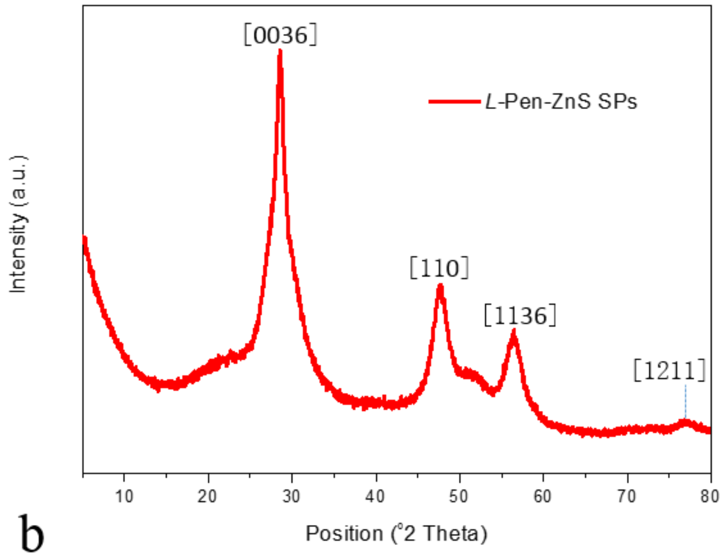

b

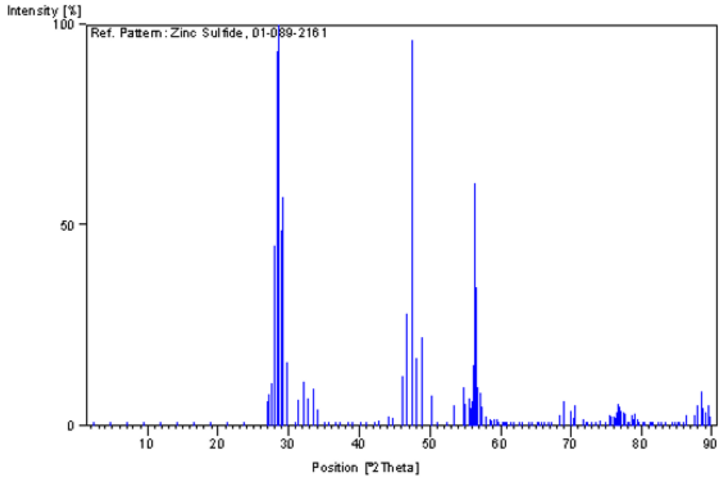

Supplementary Fig. 11 (a) XRD patterns of *L*-Pen-ZnS SPs. **(b)** The standard XRD spectra of ZnS with the PDF number of 01-089-2161.

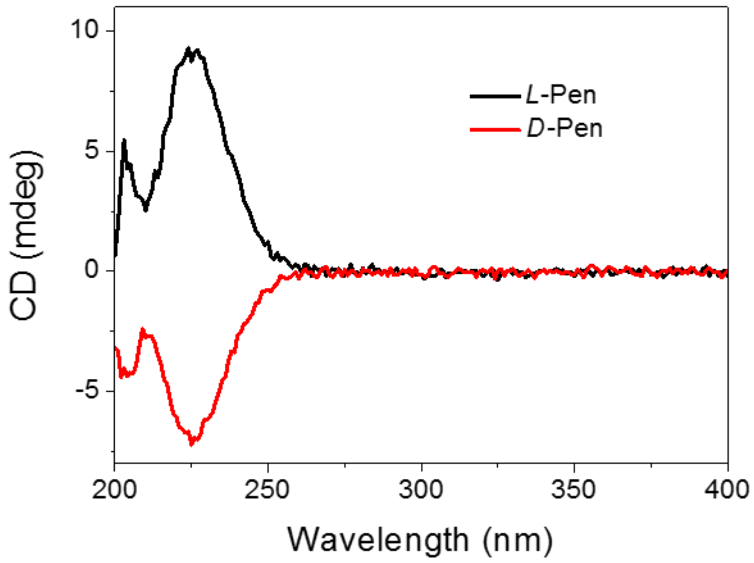

Supplementary Fig. 12 CD spectra of *L*- and *D*- Pen molecules.

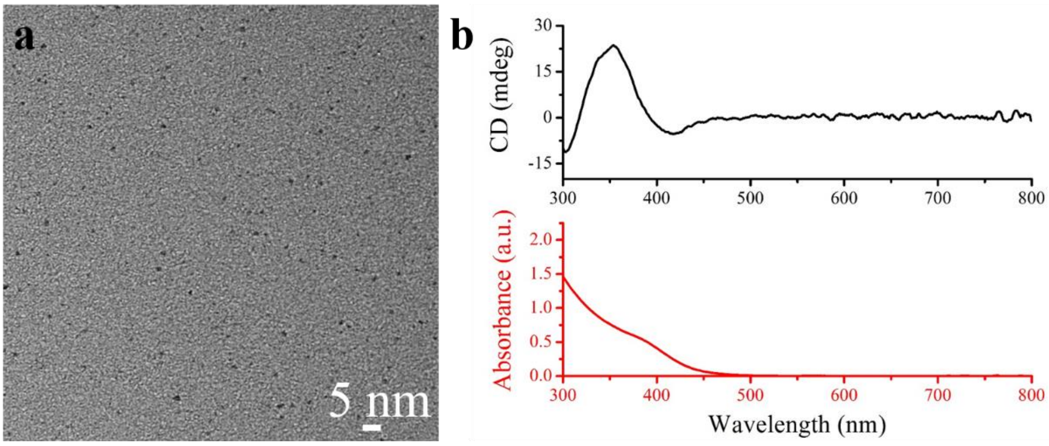

Supplementary Fig. 13 (a) TEM image and **(b)** CD and UV-Vis spectra of *L*-GSH-stabilized Au NPs.

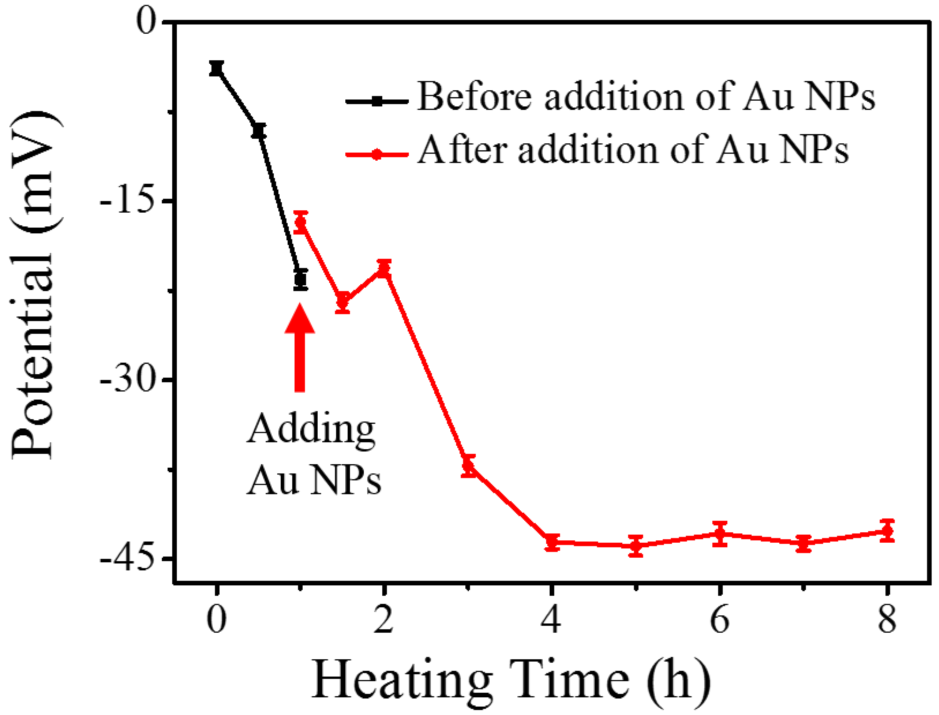

Supplementary Fig. 14 Temporal profile of ζ-potential for the assembly of chiral ZnS-Au SPs. NPs were added into the ZnS NP dispersion at 1 h heating time. The error bars represent the standard deviation of sample measurements.

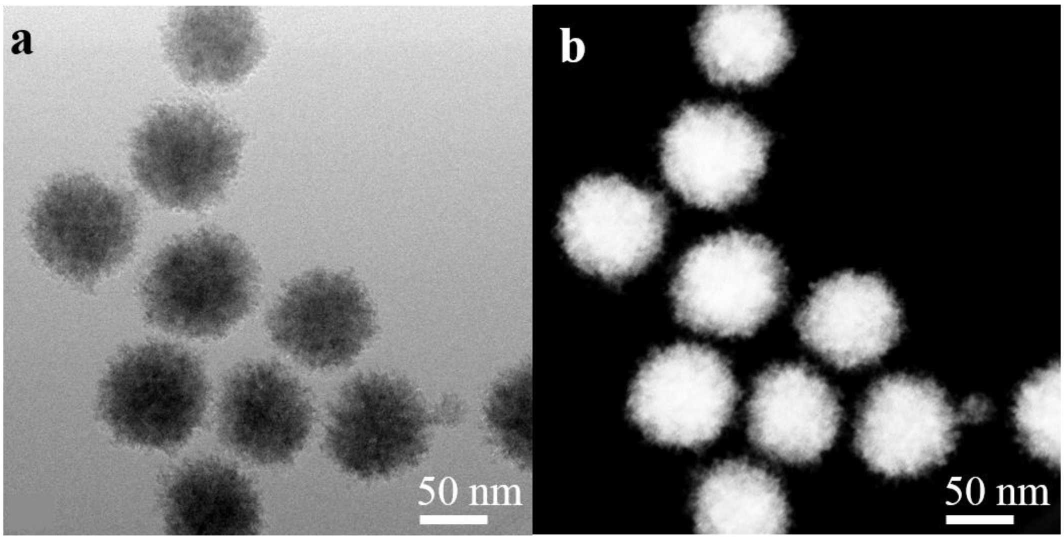

Supplementary Fig. 15 (a) Bright field- and **(b)** HAADF-STEM images of *L*-ZnS-Au SPs.

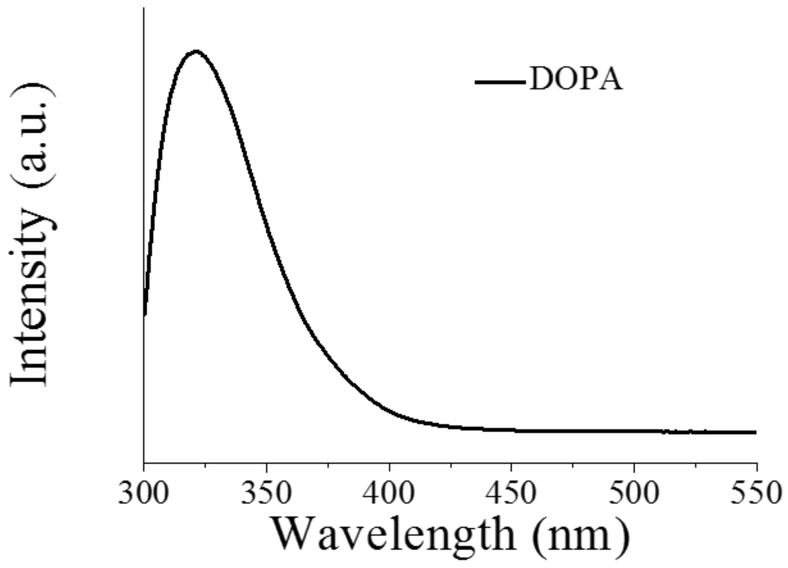

Supplementary Fig. 16 The fluorescence spectrum of DOPA.

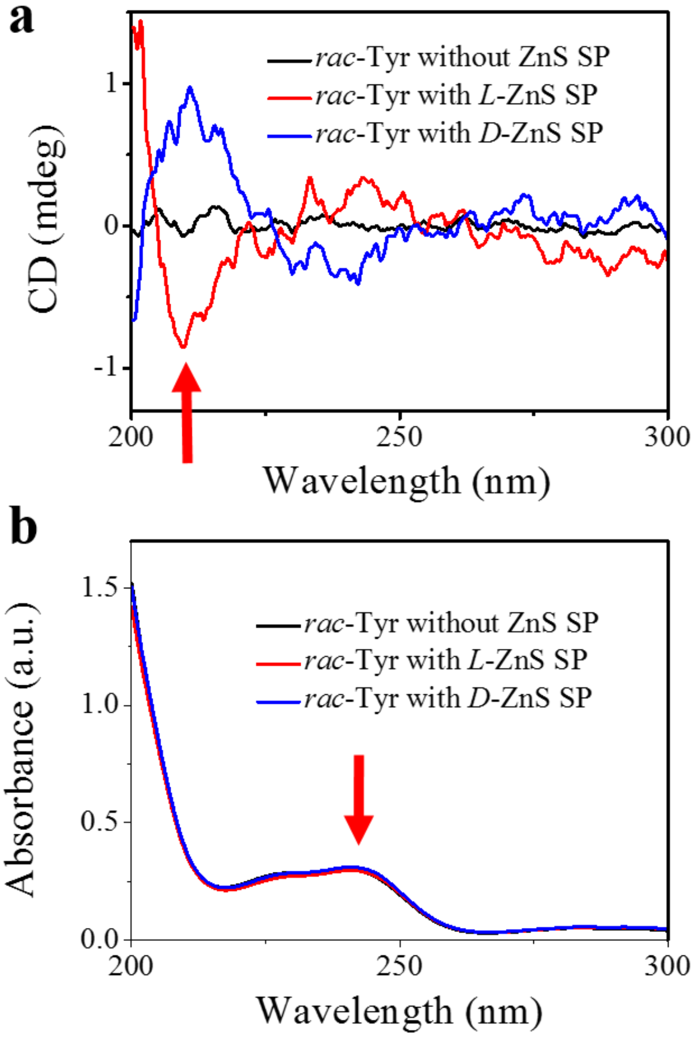
a
rac-Tyr without ZnS SP
rac-Tyr with L-ZnS SP
rac-Tyr with D-ZnS SP
CD (mdeg)
1
0
-1
200
250
300
Wavelength (nm)
b
rac-Tyr without ZnS SP
rac-Tyr with L-ZnS SP
rac-Tyr with D-ZnS SP
Absorbance (a.u.)
1.5
1.0
0.5
0.0
200
250
300
Wavelength (nm)

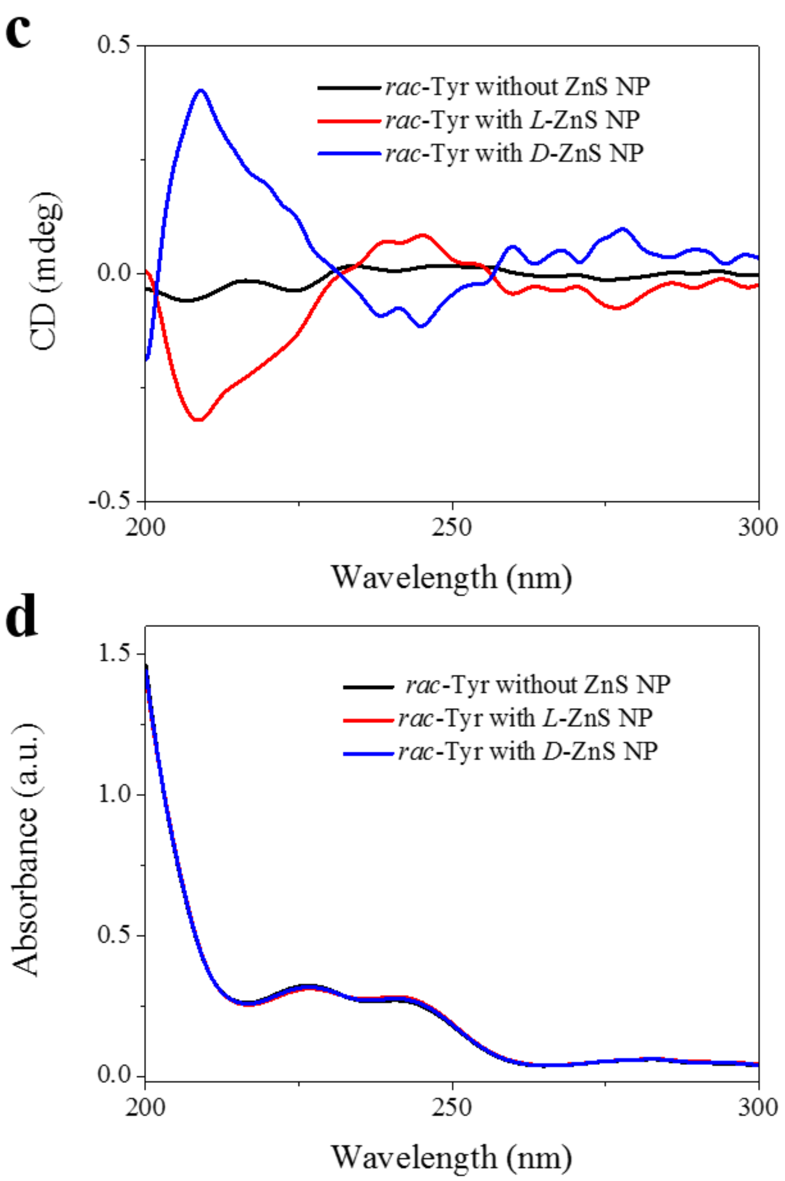

Supplementary Fig. 17 (a) CD and **(b)** UV-Vis spectra of *rac*-Tyr after incubation with, and without, *L*- and *D*-ZnS SPs. **(c)** CD and **(d)** UV-Vis spectra of *rac*-Tyr after incubation with, and without, *L*- and *D*-ZnS NPs. The CD spectra were measured for dispersions diluted to display identical UV-vis absorption at the CD peak of interests about 0.8 D.

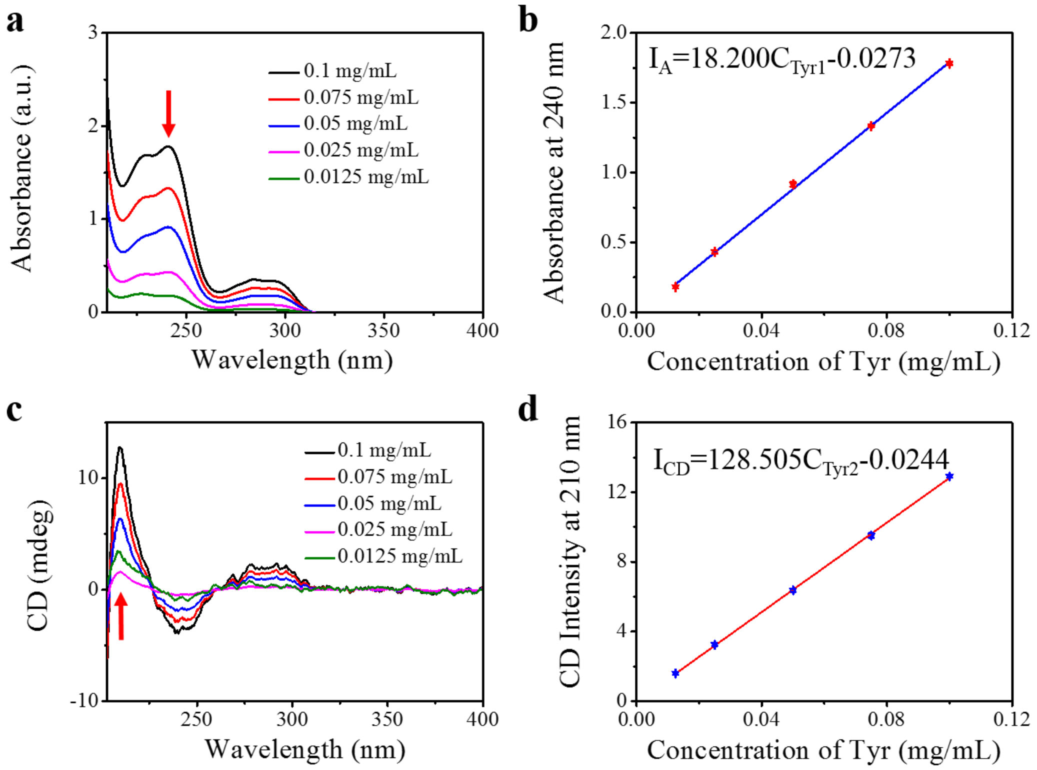

Supplementary Fig. 18 (a) UV-Vis absorption spectra of Tyr at different concentrations to establish the calibration equation for Tyr present in the supernatant. **(b)** The linear relationship between the absorption at 220 nm and the concentration of Tyr. **(c)** The CD spectra of Tyr with different concentrations. **(d)** The linear relationship between the CD amplitude at 210 nm and the concentration of Tyr. The error bars in **b** and **d** represent the standard deviation of sample measurements.

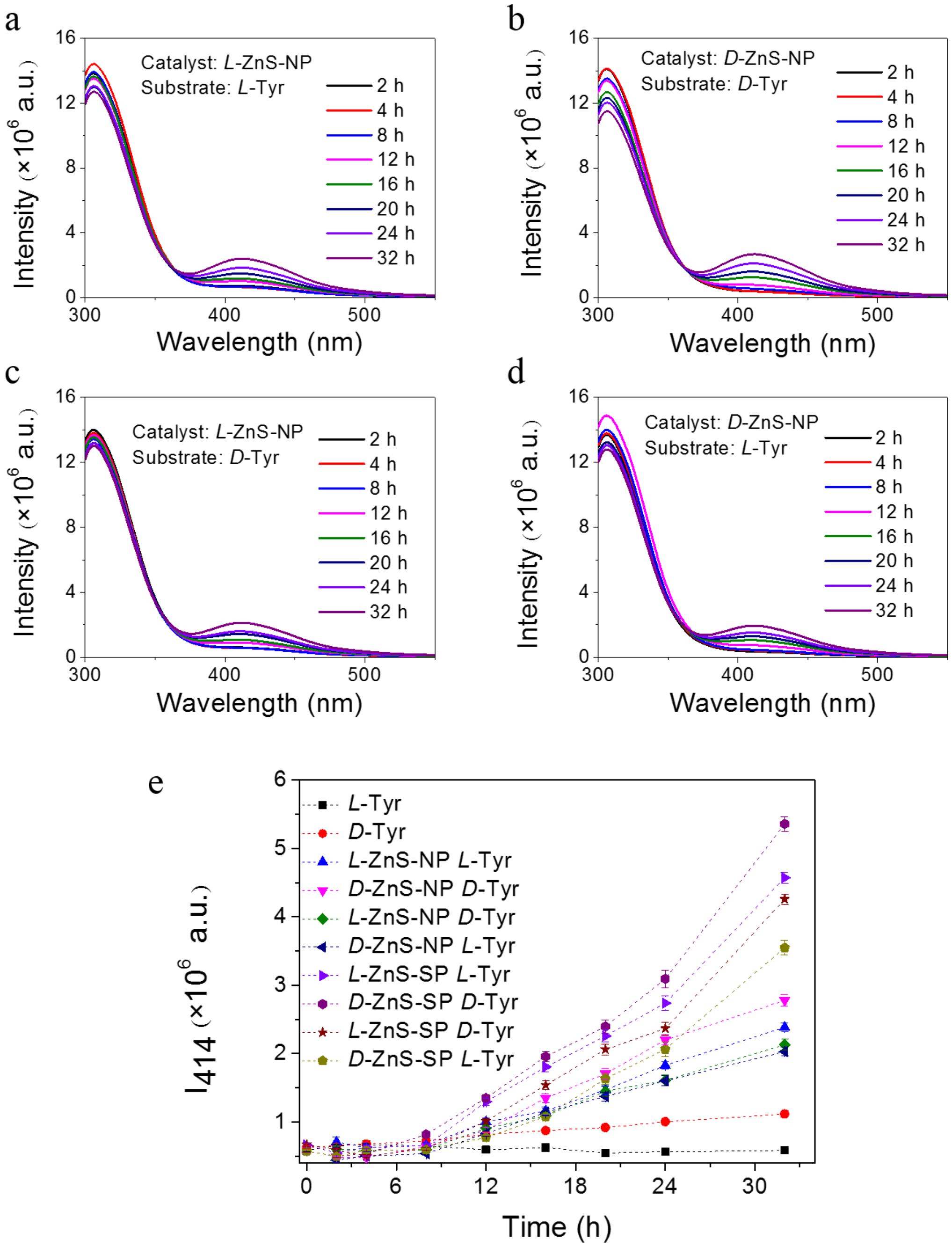

Supplementary Fig. 19 (a) *L*-Tyr with *L*-ZnS NPs. **(b)** *D*-Tyr with *D*-ZnS NPs. **(c)** *L*-Tyr with *D*-ZnS NPs. **(d)** *D*-Tyr with *L*-ZnS NPs. **(e)** The dependence of PL intensity at 414 nm on the photocatalytic reaction time with ZnS NPs and ZnS SPs of different chirality of the catalyst and the substrate. The error bars in **e** represent the standard deviation of sample measurements.

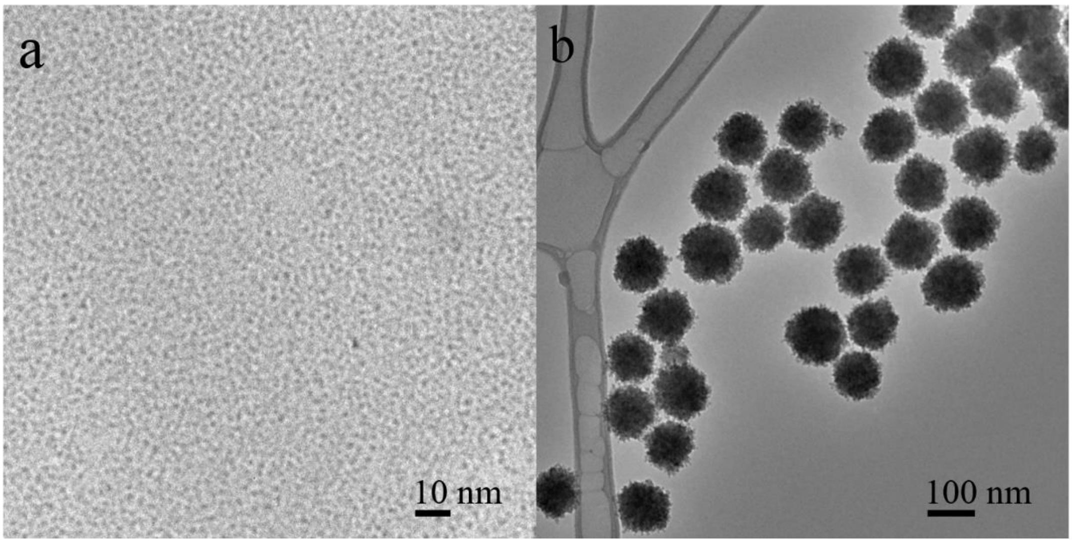

Supplementary Fig. 20 TEM images of **(a)** *L*-Pen-ZnS NPs and **(b)** *L*-Pen-ZnS SPs after photocatalysis.

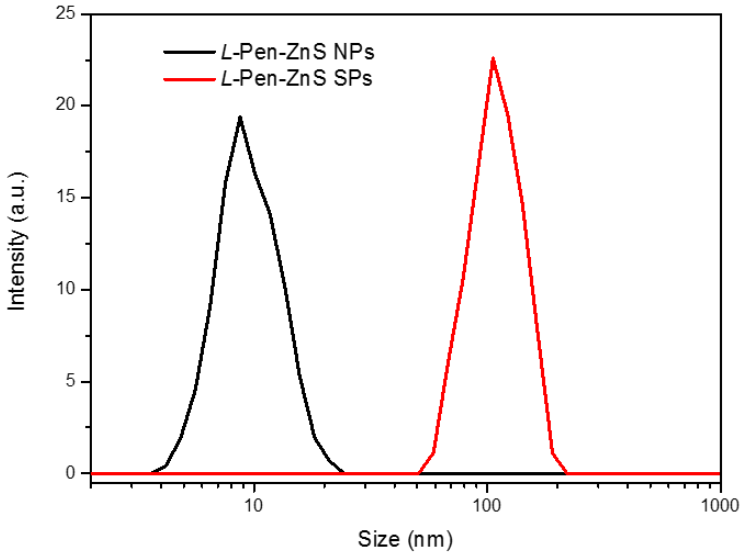

Supplementary Fig. 21 The size distribution of *L*-Pen-ZnS NPs and *L*-Pen-ZnS SPs after photocatalysis.

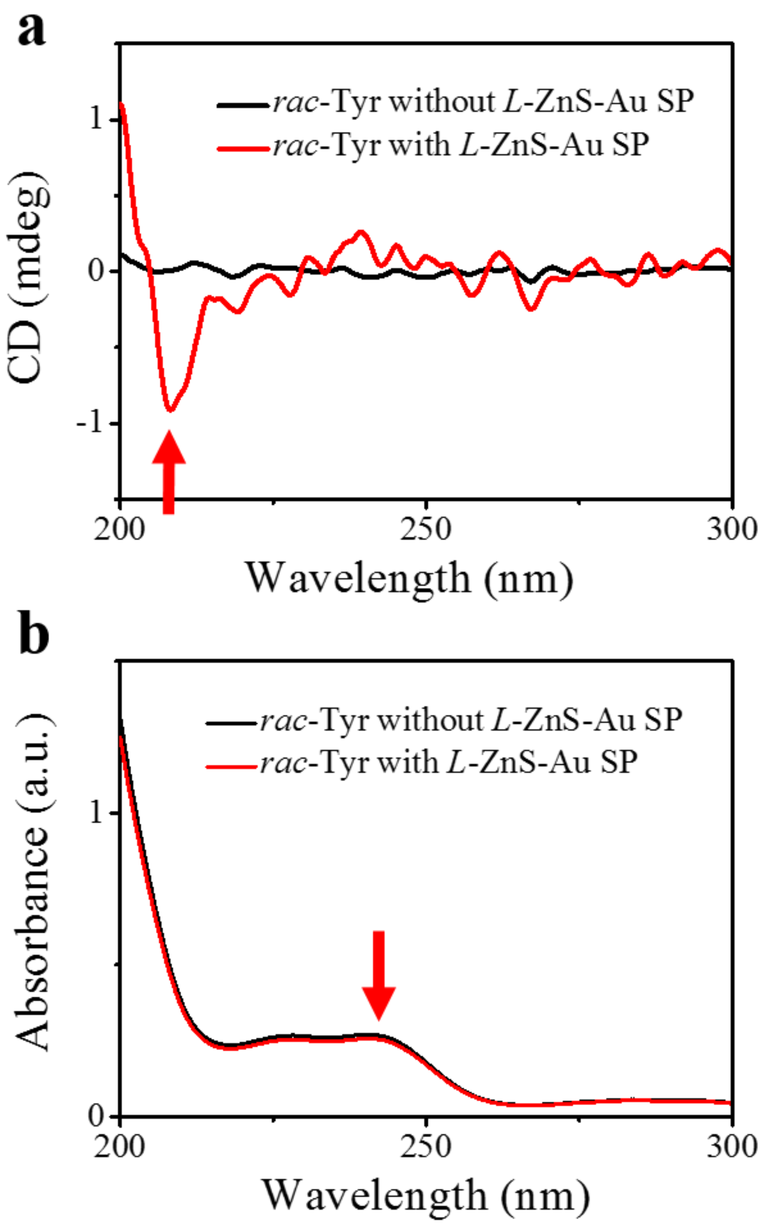

Supplementary Fig. 22 (a) CD and **(b)** UV-Vis spectra of *rac*-Tyr after incubation with and without *L*-ZnS-Au SPs. The CD spectra were measured for dispersions diluted to display identical UV-Vis absorption at the CD peak of interests about 0.8 D.

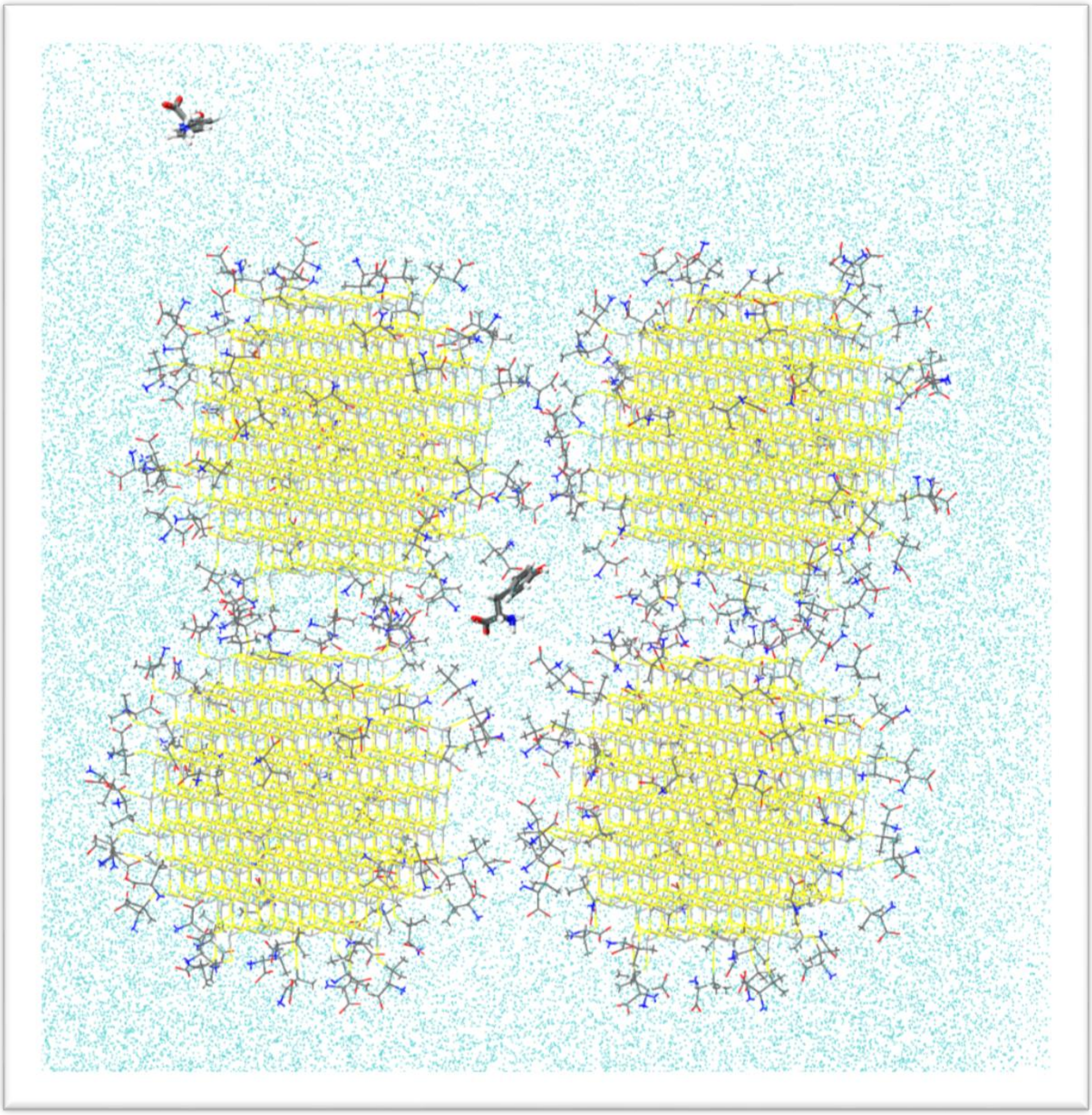

Supplementary Fig. 23. Snapshot of MD simulation with four particle assembly. *L*-Tyr (center, highlight in red circle) and L-Tyr (side, highlight in purple circle) are placed in the system of four *D*-Pen-ZnS NPs. Water molecules are represented in light blue dots.
